# A distinct epigenetic profile distinguishes stenotic from non-inflamed fibroblasts in the ileal mucosa of Crohn’s disease patients

**DOI:** 10.1371/journal.pone.0209656

**Published:** 2018-12-27

**Authors:** Andrew Y. F. Li Yim, Jessica R. de Bruyn, Nicolette W. Duijvis, Catriona Sharp, Enrico Ferrero, Wouter J. de Jonge, Manon E. Wildenberg, Marcel M. A. M. Mannens, Christianne J. Buskens, Geert R. D’Haens, Peter Henneman, Anje A. te Velde

**Affiliations:** 1 Genome Diagnostics Laboratory, Department of Clinical Genetics, Amsterdam UMC, University of Amsterdam, Amsterdam, the Netherlands; 2 Epigenetics Discovery Performance Unit, GlaxoSmithKline, Stevenage, United Kingdom; 3 Tytgat Institute for Liver and Intestinal Research, Amsterdam UMC, University of Amsterdam, Amsterdam, the Netherlands; 4 Department of Gastroenterology, Amsterdam UMC, University of Amsterdam, Amsterdam, the Netherlands; 5 Department of Surgery, Amsterdam UMC, University of Amsterdam, Amsterdam, the Netherlands; 6 Computational Biology, Target Sciences, GlaxoSmithKline, Stevenage, United Kingdom; University of Bonn, Institute of Experimental Hematology and Transfusion Medicine, GERMANY

## Abstract

**Background:**

The chronic remitting and relapsing intestinal inflammation characteristic of Crohn’s disease frequently leads to fibrosis and subsequent stenosis of the inflamed region. Approximately a third of all Crohn’s disease patients require resection at some stage in their disease course. As the pathogenesis of Crohn’s disease associated fibrosis is largely unknown, a strong necessity exists to better understand the pathophysiology thereof.

**Methods:**

In this study, we investigated changes of the DNA methylome and transcriptome of ileum-derived fibroblasts associated to the occurrence of Crohn’s disease associated fibrosis. Eighteen samples were included in a DNA methylation array and twenty-one samples were used for RNA sequencing.

**Results:**

Most differentially methylated regions and differentially expressed genes were observed when comparing stenotic with non-inflamed samples. By contrast, few differences were observed when comparing Crohn’s disease with non-Crohn’s disease, or inflamed with non-inflamed tissue. Integrative methylation and gene expression analyses revealed dysregulation of genes associated to the PRKACA and E2F1 network, which is involved in cell cycle progression, angiogenesis, epithelial to mesenchymal transition, and bile metabolism.

**Conclusion:**

Our research provides evidence that the methylome and the transcriptome are systematically dysregulated in stenosis-associated fibroblasts.

## Introduction

Crohn’s disease (CD) is a chronic condition characterized by repeated episodes of transmural inflammation in the gastrointestinal tract. While most patients display a purely inflammatory phenotype during diagnosis, fibro-stenotic complications occur in approximately a third of patients as the disease progresses. Stenosis, as a result of fibrotic tissue accumulation, is a serious complication for which no current predictors or therapeutic treatments exist, often requiring the surgical removal of the afflicted region [1]. As such, there is a strong necessity to understand the pathophysiology of CD-induced fibrosis, to allow for the development diagnostic markers and treatments.

Fibrosis is characterized by the expansion of the resident fibroblast population as well as the result of excessive production and deposition of extracellular matrix (ECM), which leads to local tissue stiffness and ultimately stenosis of the intestinal lumen [2,3]. The cause of CD-associated fibrosis is thought to be persistent tissue injury, which in turn results in the dysregulation of wound healing, unrestrained proliferation of fibroblasts, and accumulation of ECM [4]. The aberrant wound healing process, in combination with the accumulation of ECM, eventually leads to fibrosis, which may become self-propagating [5].

Genome-wide association studies (GWAS) have identified over 200 loci associated to inflammatory bowel disease (IBD) [6], many of which either associate to several genes or are not annotated. Despite the extensive research into the genetic component underlying IBD, it is estimated that common genetic variants explain only 20% of the estimated heritability (30–50%) [6–8]. Therefore, IBD has been classified as a complex disease, the etiology of which is likely a combination of genetic [9], epigenetic [10–13] and other environmental factors [7,14].

Epigenetic modifications affect the readability and transcription of the genome without changing the actual sequence. The dynamic behavior of the epigenetic landscape is partially a response to environmental influences, suggesting a putative link between CD-associated fibrosis and the environment [15,16]. One of the most widely investigated epigenetic modifications is cytosine methylation, with previous epigenome-wide association studies (EWAS) investigating CD and IBD in leukocytes reporting many differentially methylated loci in genes that are part of inflammatory and immune related pathways [17–22]. Despite the many statistically significant loci, the actual differences in methylation were often small (<20% mean methylation difference). In contrast, samples derived from the ileocolon revealed larger changes in DNA methylation [23,24], suggesting that the methylome is more profoundly affected at the sites of inflammation rather than in peripheral tissues. Here, we investigated whether fibro-stenosis manifests itself within the DNA methylome and transcriptome to identify putative drivers of the fibro-stenotic phenotype by comparing CD and non-CD, as well as among different stages of CD. In particular, we chose to investigate ileal fibroblasts, as fibro-stenosis with complications occur primarily in the terminal ileum of CD patients [25].

## Materials and methods

### Patient and sample selection

Fibroblasts were isolated from the mucosa of terminal ileal tissue of CD patients undergoing ileocecal resection surgery due to therapy-refractory disease at the Academic Medical Center in Amsterdam, the Netherlands (Table 1). The CD fibroblasts were isolated from the mucosa of the ileum, which appeared macroscopically normal (‘non-inflamed’; NINF), inflamed (INF), or stenotic (STEN), as determined by the treating surgeon and confirmed by qualified researchers in the laboratory. The control fibroblasts were obtained from patients without a history of IBD who underwent resection for colon ascendance malignancies. Fibroblasts were isolated from mucosal tissue at a distance of at least 10 cm from neoplastic lesions to minimize tumor involvement [26]. In addition to the phenotypic appearance of the sample of origin, additional metadata such as age, sex, current treatment, smoking status, and Montreal classification [27] were documented as well (Table 1).

**Table 1:** Patient characteristics.

**A**					
**DNA**	**CD (n = 7)**				**non-CD (n = 3)**
**Age at time of surgery** **(mean ± standard deviation)**	29.7 (± 10.3)				46 (± 22.6)
**Sex (% males; n)**	71% (5)				66% (2)
**Current treatment with biological agent (% yes; n)**	71% (5)				-
**Current smokers (% yes; n)**	28% (2)				0% (0)
**Montreal classification (%; n)**					-
**A1 / A2 / A3**	14% (1)	86% (6)	0%		
**L1 / L2 / L3 / L4**	57% (4)	0%	43% (3)	0%	
**B1 / B2 / B3 / B2+B3**	14% (1)	29% (2)	14% (1)	43% (3)	
**P**	29% (2)				
**B**					
**RNA**	**CD (n = 10)**				**non-CD (n = 4)**
**Age at time of surgery (mean ± standard deviation)**	35.7 (± 14.4)				54.5 (± 18.3)
**Sex (% males; n)**	60% (6)				75% (3)
**Current treatment with biological agent (% yes; n)**	60% (6)				-
**Current smokers (% yes; n)**	20% (2)				0% (0)
**Montreal classification (%; n)**					-
**A1 / A2 / A3**	10% (1)	90% (9)	0%		
**L1 / L2 / L3 / L4**	50% (5)	0%	50% (5)	0%	
**B1 / B2 / B3 / B2+3**	20% (2)	30% (3)	20% (2)	30% (3)	
**P**	10% (1)				

Overview of the samples used in the DNA methylation and gene expression experiments. The Montreal classification represents a scale which is used to classify disease severity of IBD.

An overview of all the samples included in the DNA methylation and the RNA sequencing experiments is provided in S1 Table. In short, the DNA methylation experiment was performed on 18 samples isolated from 10 unique patients (7 NINF from 4 patients, 2 INF from 2 patients, 4 STEN from 3 patients, and 5 non-CD from 3 control patients). The RNA sequencing experiment was performed on 21 samples isolated from 14 unique patients (6 NINF from 6 patients, 4 INF from 4 patients, 5 STEN from 4 patients, and 6 non-CD from the 4 control patients). Of the samples used for DNA methylation and RNA sequencing, 9 from 6 unique patients were present in both experiments.

### Fibroblast isolation

Mucosal samples were thoroughly washed in repeated cycles of ice-cold phosphate buffered saline (PBS) supplemented with 1% penicillin/streptomycin and 40 μg/mL gentamicin (PGA). The mucosa was then finely cut and placed in full-grown RPMI 1640 culture medium (Invitrogen) supplemented with 1.5 mg/mL collagenase A (Roche, Germany) and minced using the Gentlemacs Dissociator (Miltenyi Biotec, Leiden, the Netherlands). After 60 minutes of incubation at 37°C, the Gentlemacs Dissociator was used a second time for further dissociation. Cells were transferred to tubes and washed extensively with PGA, after which the cells were plated and cultured at 37°C in RPMI 1640 with 10% FCS, 1% penicillin/streptomycin, 1% L-glutamin, 40 μg/mL gentamicin (Lonza, Leusden, the Netherlands) and 0.025 μg/mL amphotericin B (Gibco, Rockford, IL) [28].

### Immunohistochemistry

Tissue was fixed in 4% paraformaldehyde, embedded in paraffin and sectioned at 4 micrometer. Sections deparaffinized, rehydrated and immersed in 0.3% H_2_O_2_ and methanol for 30 minutes. Antigen-retrieval was performed using Tris-EDTA (CD3) or NaCi (collagen type I) after which slides were blocked with PBS, 0.1% Triton X-100 and 1% bovine serum albumin (PBT) for 30 minutes. Slides were subsequently incubated with the primary antibody overnight at 4°C (rabbit monoclonal anti-human CD3 [clone SP7, Thermo Scientific] and goat polyclonal collagen I [Southern Biotech, Birmingham, AL, USA]) in PBT, after which they were incubated with Brightvision [Immunologic, Duiven, the Netherlands], developed using DAP, counterstained with haematoxylin and mounted.

### Passaging and harvesting of fibroblasts

After 24 hours of culturing, fibroblast cells were adhered to the culture plates and the RPMI medium was refreshed to wash away debris, dead-, non-adherent- and non-fibroblast cells. After the cells reached a minimum of 80% confluency, they were washed with HBSS (Lonza BioWhittaker, Switzerland) and detached from the culture plates through a ten-minute trypsin wash (10x diluted in HBSS) at 37°C. The fibroblasts were then plated in T25 flasks, after which samples in later passages were transferred to T75 flasks (VWR, Pennsylvania, USA; Tissue Culture Flasks 25 cm2 & 75 cm2) to allow for further expansion. During passages 1 through 5, cells were harvested for downstream DNA and RNA analysis. For DNA isolation, fibroblasts were stored in PBS at -80°C, while cells for RNA isolation were stored in RNAlater (Thermo Fisher Scientific, Waltham, Massachusetts, USA) at 4°C overnight, after which the samples were transferred to -80°C as per manufacturer’s protocol. Eighteen DNA samples were isolated from 10 unique patients for the methylation experiment, whereas 25 RNA samples were isolated from 15 unique patients for the RNA sequencing experiment.

### Methylation analysis

Analysis of the methylation data was performed in the R statistical programming environment [29] (v3.3.2) using the Bioconductor (v3.4) packages minfi [30] (v1.24.0) for import, MethylAid [31] (v1.12.0) for quality control, functional normalization for normalization [32], limma [33,34] (v3.34.9) for linear regressions to find differentially methylated positions (DMPs), and DMRcate [35] (v1.14.0) to find differentially methylated regions (DMRs). Regions were found to be differentially methylated if it contained at least 3 CpG-probes. Annotation of the DMRs was performed using ChIPSeeker [36], which associates regions of interest to genes according to their location relative to the nearest transcription start site (TSS). Samples were included in this study if they passed the detection *p*-value quality control and were not an outlier according to the principal component analysis. Probes were excluded from the analyses if their CpG of interest overlapped with a known SNP (minor allele frequency > 0.05), if the probe was found to be promiscuous [37]. Furthermore, all probes associating to the sex chromosomes were removed due to the mixed-sex cohort. Statistical significance was defined as DMRs that had a Stouffer transformed Benjamini-Hochberg (BH) adjusted *p*-value below 0.05. Linear regressions were performed using the M-values, whereas the Beta-values were utilized for the visualization [38]. Covariates in the DMP and DMR analyses included passage number, age, sex, and medication usage (azathioprine, purinethol, adalimumab, or infliximab). An additional random effects component (‘blocking’) for patient source was utilized to correct for samples obtained from the same patient. Plots were made using ggplot2 (v2.2.1) [39].

### Metadata anonymous sample

Due to the anonymity of one control sample, no metadata was available. The age and sex were therefore estimated using the DNA methylation and gene expression data. The age was estimated using 353 age-related CpG sites as described by Horvath [40], after which the age-related CpG sites were removed to prevent any confounding of downstream analyses. The sex was estimated based on the presence of a methylation signal of probes located on chromosome Y and was subsequently confirmed by the presence of gene expression signals coming from chromosome Y.

### Gene expression analysis

RNA was isolated from the fibroblasts using the RNAeasy mini kit (Qiagen) and the quality was measured using the BioAnalyzer, whereby RNA samples with a RIN score of 8 or higher were taken for further analysis. The NEBNext Ultra Directional RNA Library Prep Kit (New England BioLabs) was used for mRNA isolation, cDNA generation, and sequencing adapter ligation. Next generation sequencing of the resulting cDNA libraries was performed on an Illumina NextSeq500 at a coverage of 10M reads per sample. The preparation and sequencing of the RNA was performed at GenomeScan B.V. in Leiden, the Netherlands (ISO/IEC 17025 (L518) accredited).

For the differential gene expression analysis, raw sequences were merged per sample and checked for quality using FastQC [41] (v1.11.7) and MultiQC (v1.4). The sequences were then aligned to the reference genome GRCh38 using STAR [42] (v2.5), and sorted and converted into binary files using SAMtools [43] (v1.2). Reads were counted and annotated using the Ensembl annotations (v90) using the featureCounts module in the Subread package [44] (v1.28). Subsequent normalization and differential gene expression analysis was performed using DESeq2 [45] (v1.18.1). Similar to the methylation analysis, we corrected for passage number, age, sex, and medication usage (azathioprine, purinethol, adalimumab, or infliximab). Genes were excluded if the count per million (CPM) had was less than 1 for more than 11 samples. Plots were made using ggplot2 [39].

### Integrative methylation-expression analysis

Genes that were differentially expressed and differentially methylated (DMEGs) were extracted and subjected to an expression quantitative trait methylation analysis (eQTM). In short DMRs were correlated with the log transformed count data obtained from the expression analysis for the 9 samples that were analyzed in both the methylation and the expression analysis (S1 Table). The mean Beta value per DMR was calculated per sample after which Pearson correlations were calculated for each DMR-gene pair. The 95% confidence intervals were calculated through 10000 bootstraps and *p*-values were calculated by comparing our observed correlation coefficient against a permutation-based null-distribution. The null-distribution was generated by randomly generating 10000 regions with equal numbers of CpGs as the observed DMR (‘null-DMRs’). Resulting plots were generated using Gviz (v1.22.3) [46] and ggplot2 [39].

### Pathway enrichment and gene set overrepresentation analyses

As DMRs, DEGs and DMEGs represent different entities, different tools were used for pathway enrichment and gene set overrepresentation analyses. DMRs represent genomic regions and thus we performed pathway enrichment analyses using the ChIP-Enrich (v2.2.0) package to account for the length of the DMR [47]. Conversely, DESeq2 normalizes for the locus length during differential expression analysis, which does not need to be corrected for during the enrichment analysis. Instead, pathway enrichment analyses of the DEGs were performed using the camera function from limma [48], which performs a competitive gene set test to account for inter-gene correlation. For the enrichment analyses of the DMRs and the DEGs, the Reactome [49] and Kyoto Encyclopedia of Genes and Genomes (KEGG) [50] pathway databases were used as reference datasets. To obtain a summary of the observed DMEGs regardless of the direction of methylation or expression we used the ConsensusPath database webtool, where we performed gene set overrepresentation analyses against the network neighborhood-based entity sets (NESTs), all available pathway gene sets, and the protein complex-based gene sets [51].

### Data availability

Both the raw and the processed data have been made publicly available in the GEO repository GSE99788 and GSE99816 for the DNA methylation and RNA sequencing experiments, respectively. The scripts used for data analysis are located at https://github.com/ND91/PRJ0000003_LiYim2018.

### Ethical considerations

Part of the patient samples were acquired from the surgical department with signed informed consent prior to surgery, as reviewed and approved by the institutional ethics committee at the Academic Medical Center Amsterdam (“Medisch Ethische Toetsings Commissie AMC”, reference: METC #2014_178). Other patient samples were collected from the pathology department and were granted a waiver by the institutional ethics committee (reference: W12_216 # 12.17.0246).

## Results

### Genome-wide differences in methylation when comparing stenotic with non-inflamed tissue

**Fig 1:**
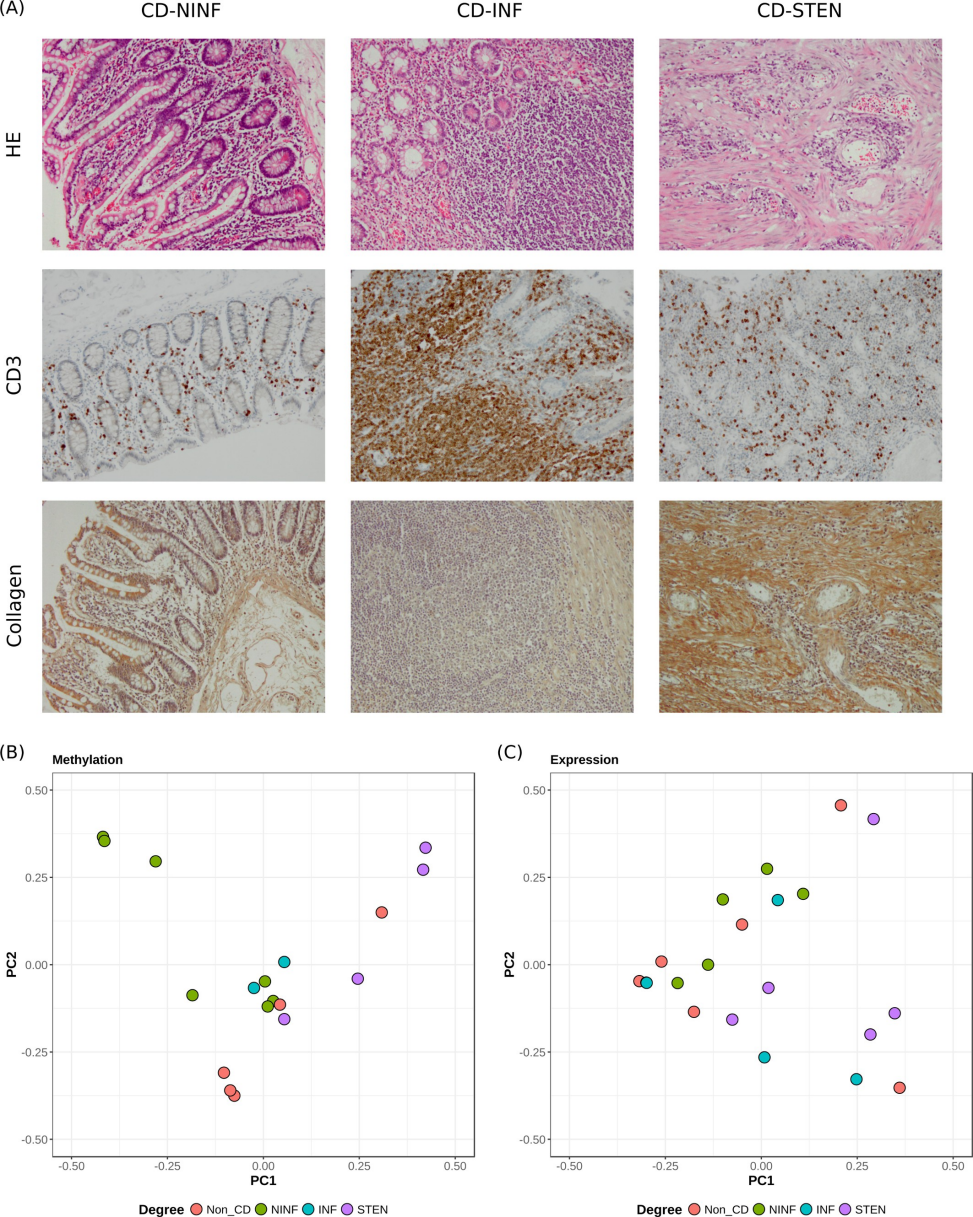
Immunohistochemical staining and principal component analyses. (A) General staining for haematoxylin and eosin (HE) and specific staining for CD3 and collagen. Principal components 1 and 2 for (B) DNA methylation, and (C) gene expression.

Ileal material was obtained from CD and non-CD patients undergoing resection after which it was macroscopically classified into non-inflamed (NINF), inflamed (INF), and stenotic (STEN) tissue. Immunohistochemical staining for CD3 showed that INF tissue displayed more CD3+ cells relative to the NINF and the STEN tissue samples. Furthermore, general staining for heamatoxylin and eosin (HE) as well as specific staining for collagen indicated that the STEN samples are mainly fibrotic in nature as shown by extensive collagen deposition (Fig 1A). It should be noted that as the STEN samples are obtained from an affected area, levels of CD3 infiltration are increased relative to NINF samples, albeit far below the levels seen in the INF samples (Fig 1A). Mucosal fibroblasts were subsequently extracted from the resection material and cultured to prevent the growth of other cell types.

We were primarily interested in differences in methylation and expression between (i) CD and non-CD patients, as well as the comparisons within CD patients: (ii) INF vs NINF and (iii) STEN vs NINF. As the fibroblasts had been cultured and grown to different passages, we investigated whether any effect was evident at a genome-wide level by means of principal component analysis (PCA). We observed limited to no correlation between passage and the first three principal components for both the methylation and the expression data (r^2^_passage_ < 0.3), suggesting that passage does not affect the methylation or gene expression overall (S1 Fig). In addition, we correlated the principal components with the other factors: CD degree, age, sex, smoker status and medication usage. Limited correlation was observed between medication usage and the first three principal components (r^2^_medication_ < 0.2). Sex and age however were moderately correlated with the second principal component for the methylation data (r^2^_sex_ = 0.41; r^2^_age_ = 0.53) and the moderately with the first principal component for the expression data (r^2^_sex_ = -0.05; r^2^_age_ = 0.26). Interestingly, the strongest correlation with the first principal component for the methylation data was observed for NINF < INF < STEN (r^2^_degree CD_ = 0.46). Visualization of the first two principal components for the methylation data revealed a clear distinction between STEN and NINF tissue (Fig 1B), suggesting genome-wide differences in methylation. However, visualization of the first two principal components of the gene expression data did not show any separation among the different degrees of CD (Fig 1C). To mitigate the effect of the passage, sex, age and medication usage, they were included as covariates in the downstream linear regression analyses.

### Stenosis-associated differentially methylated regions affect tissue remodeling processes

**Fig 2:**
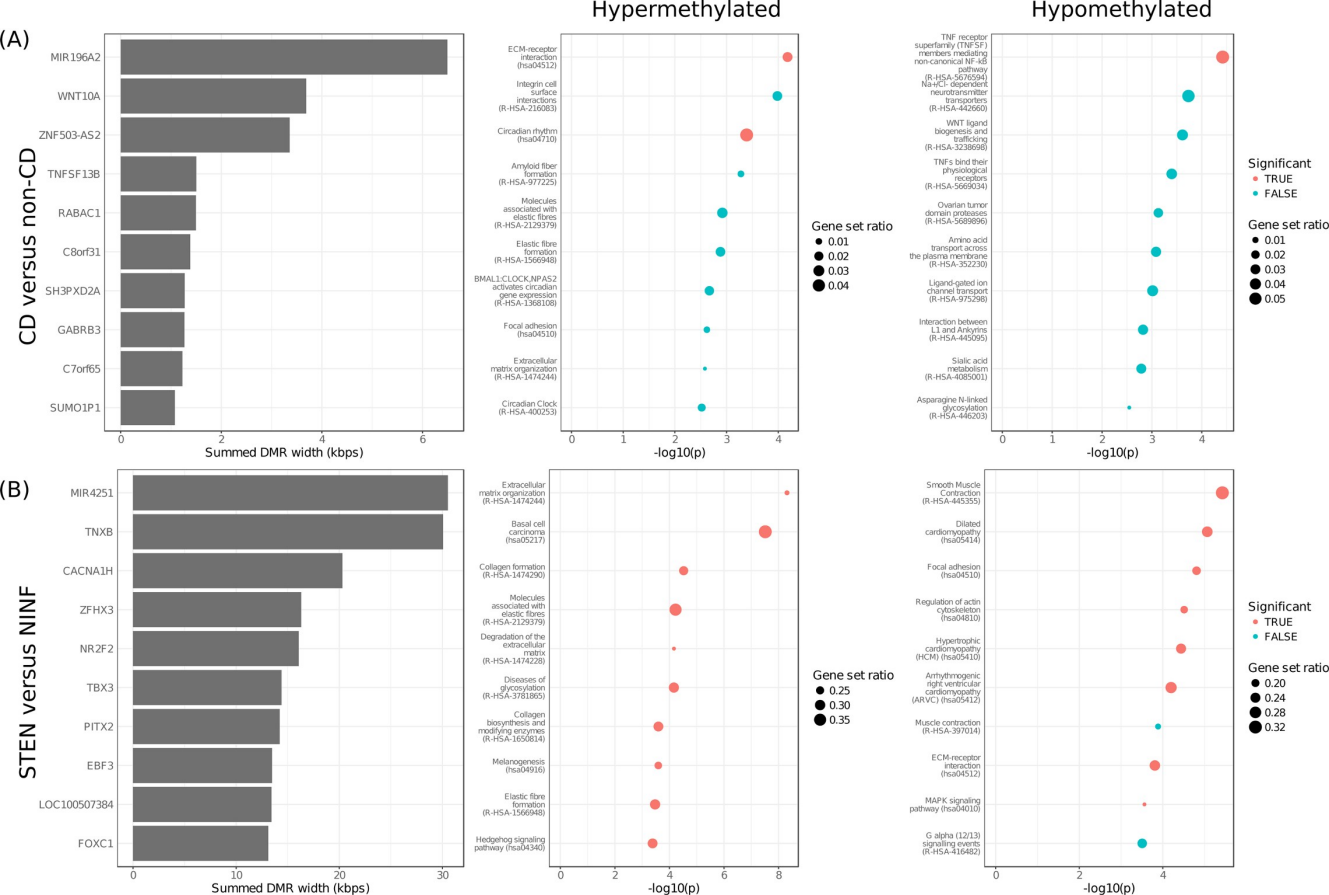
Enrichment analyses of the DMRs. Enrichment analyses results of the DMRs when comparing (A) CD with non-CD and (B) STEN with NINF. From left to right: Top 10 genes with the longest aggregated DMR, top 10 enriched pathways for the hypermethylated DMRs, and top 10 enriched pathways for the hypomethylated DMRs.

Systematically searching for differentially methylated positions (DMPs) yielded 44, 84621, and 123877 DMPs when comparing CD with non-CD, CD INF with CD NINF and CD STEN with CD NINF, respectively (Table 2) In agreement with the PCA, most DMPs were found when comparing STEN with NINF fibroblasts (S2 Fig). We reasoned that continuous regions of differential methylation (DMRs) are more likely biologically relevant and therefore investigated the presence of DMRs. In total, 38, 4, and 4883 DMRs were found for the comparisons CD with non-CD, INF with NINF, and STEN with NINF, respectively (Tables 2 and S2). For the CD with non-CD and the STEN with NINF comparisons we observed several genes to be affected by multiple DMRs. By summing the lengths of the DMRs per gene and then sorting them, we observed long regions (>3 kbps) of differential methylation for Wnt family member 10A (*WNT10A*) and Tenascin-X (*TNXB*) when comparing CD with non-CD and STEN with NINF, respectively (Fig 2A and 2B).

**Table 2:** Results overview.

Comparison	DMPs	DMRs	DEGs
**CD vs Non-CD**	44	38	11
**CD-INF vs CD-NINF**	84,621	4	0
**CD-STEN vs CD-NINF**	123,877	4,883	261

A summary of the results found from the methylation (DMPs and DMRs) and gene expression (DEGs) analyses.

To understand the functional implication, we performed pathway enrichment analyses on hypo- and hypermethylated DMRs separately. As only few DMRs were found for the comparison INF vs NINF, we performed enrichment analyses only on the DMRs found when comparing CD with non-CD and STEN with NINF. The CD vs non-CD DMRs revealed enriched hypermethylation for ECM-processing, whereas tumor necrosis factor (TNF) receptor superfamily members were enriched for hypomethylation (Fig 2A and S3 Table). STEN vs NINF hypermethylated DMRs were enriched for various pathways associated to extracellular matrix and collagen biosynthesis, whereas hypomethylated DMRs were enriched for smooth muscle contraction, focal adhesion and various other signaling pathways (Fig 2B and S3 Table).

### Downregulation of genes involved in extracellular matrix processes associated to stenosis

Following the methylation analyses, we sought to investigate what differences could be observed at the level of gene expression. Analysis of the gene expression profiles yielded fewer results than the methylation analyses. Overall, we observed 11, 0 and 261 differentially expressed genes (DEGs) when comparing CD with non-CD, INF with NINF, and STEN with NINF, respectively (Tables 2 and S4). Like the methylation data, most DEGs were found when comparing STEN with NINF. Overall, we observed a general downregulation of the DEGs as visible from the volcano plot (S3 Fig).

**Fig 3:**
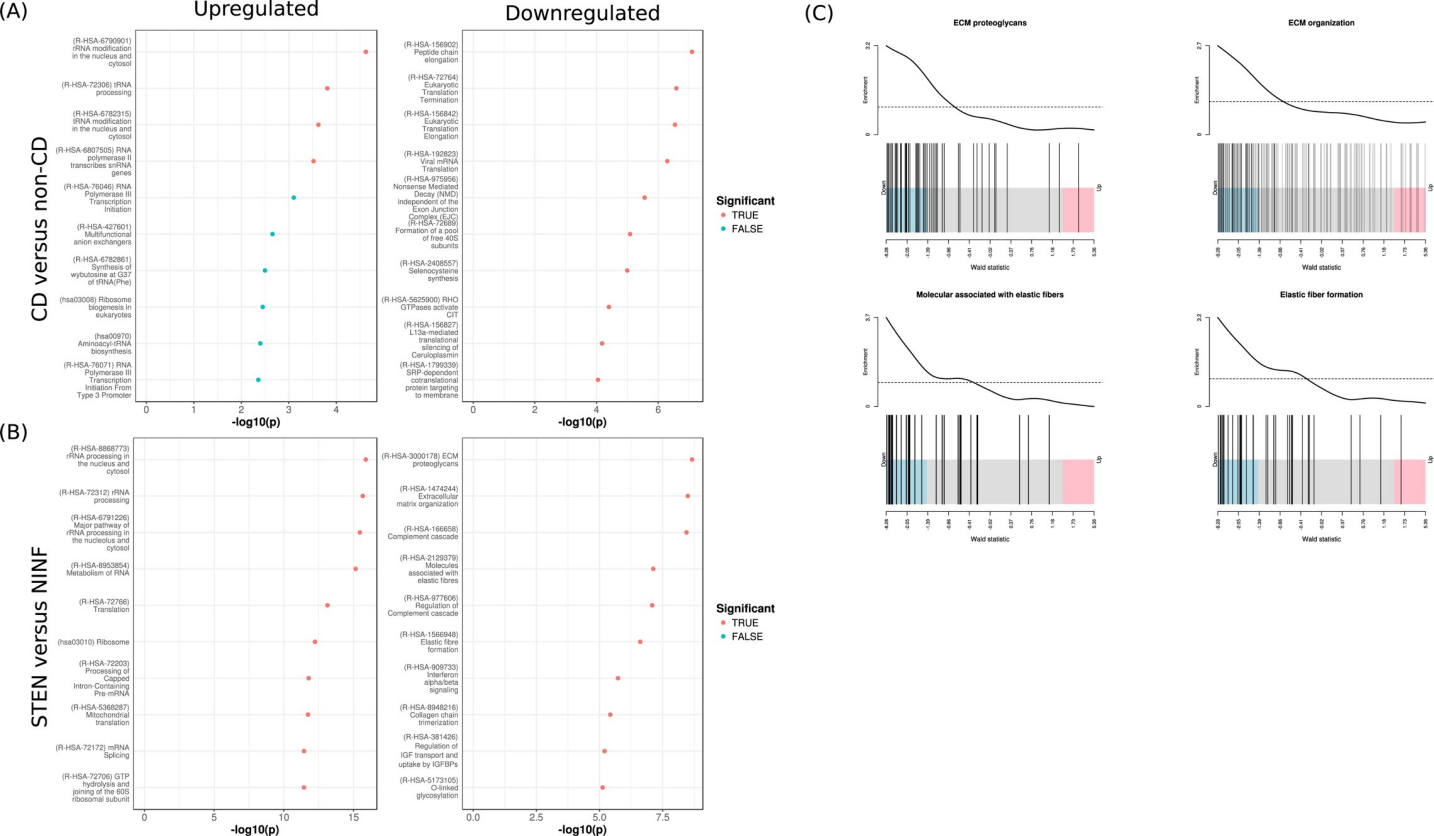
Enrichment analyses of the DEGs. Enrichment analyses results when comparing (A) CD with non-CD and (B) STEN with NINF. Top 10 enriched pathways for up- and downregulated genes on the left and right, respectively. (C) Barcode plots depicting the gene enrichment for ECM-associated pathways: “Molecules associated with elastic fibers”, “Elastic fiber formation”, “ECM proteoglycans”, and “Extracellular matrix organization”.

Pathway analysis of the transcriptional changes when comparing CD with non-CD genes revealed upregulation of genes associated with RNA processing and downregulation of genes associated with translation (Fig 3A). Transcriptional changes when comparing STEN with NINF indicated upregulation of genes associated with RNA processing and downregulation of genes associated to elastic fiber formation, ECM proteoglycans, and extracellular matrix organization (Fig 3B and S5 Table).

### Integrative methylation and expression analysis suggests concordant dysregulation of genes associated to PRKACA and E2F1

**Fig 4:**
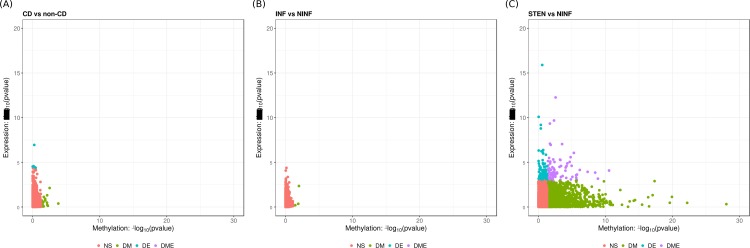
Methylation-expression integration. Overlap of -log_10_(*p*-value) of the results observed for the DMR analysis (x-axis) and the DE analysis (y-axis) for (A) CD vs non-CD, (B) INF vs NINF, and (C) STEN vs NINF. Colors represent statistical significance: red represents non-significant (‘NS’), green represents differentially methylated (‘DM’), blue represents differentially expressed (‘DE’), and purple represents differentially methylated and expressed (‘DME’).

We subsequently integrated the DNA methylation and gene expression data by searching for genes that were differentially expressed and differentially methylated, and quantified the correlation through an expression quantitative trait methylation analysis (eQTM; S6 Table). No overlap was observed when comparing CD with non-CD or INF with NINF (Fig 4). When comparing STEN with NINF, we observed 76 DEGs that were associated to a DMR as well (DMEGs). Overrepresentation analyses of the DMEGs corroborated our the observations on the methylation and expression data alone with significant overrepresentation was for genes interacting with elastin, as well as genes encoding for proteins that are part of the transforming growth factor β (TGFβ)-, and fibroblast growth factor receptor (FGF:FGFR) protein complexes (S7 Table).

**Fig 5:**
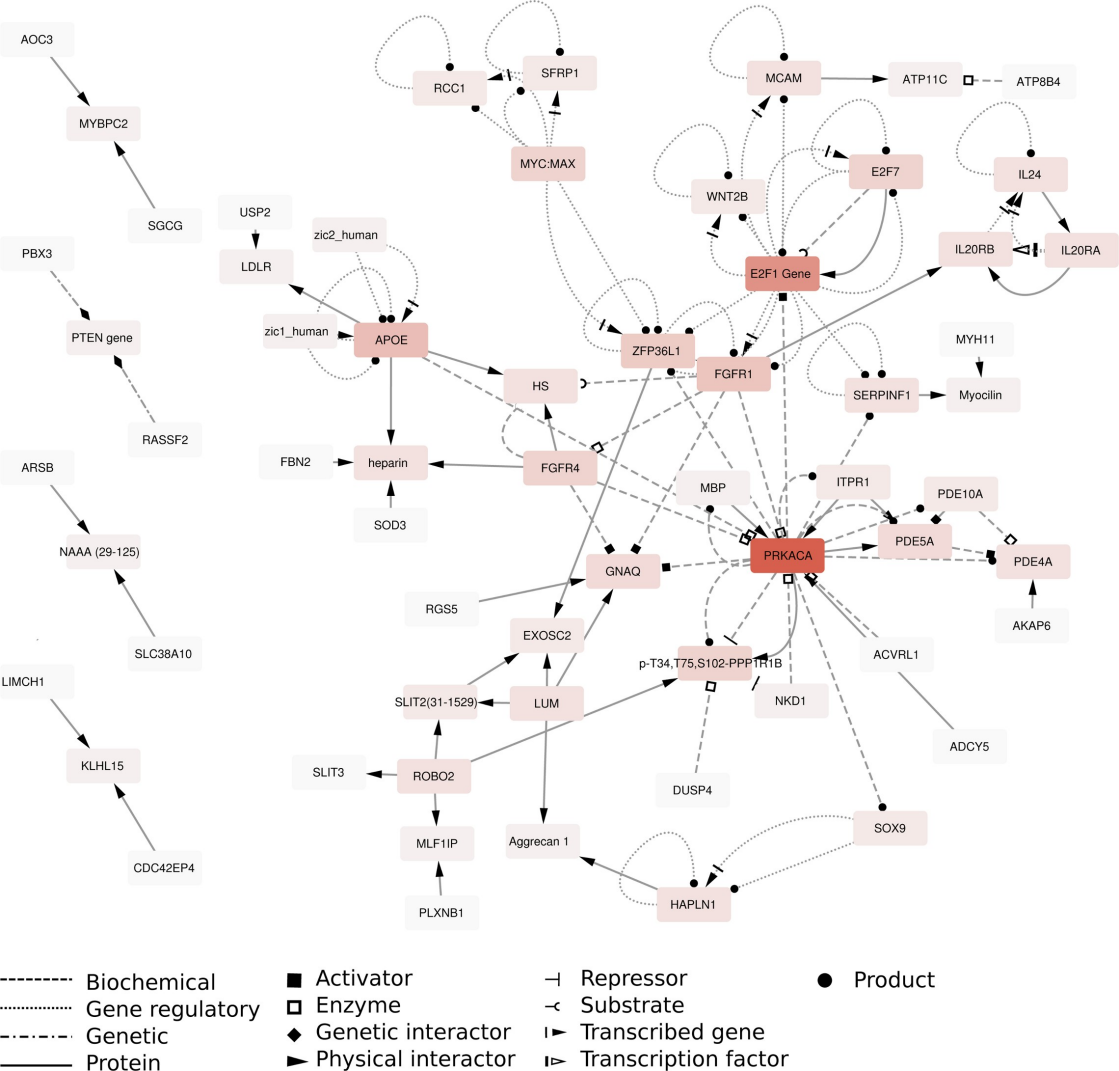
Induced network analysis. Induced network analysis of the DMEGs generated by the ConsensusPathDB. Edges represent the relationships between genes, which are represented as nodes. The intensity of the color of a node is proportional to the number of interactions known for that entity. Dashed lines represents biochemical interactions, dotted lines represents gene regulatory interactions, dashed-dotted lines represents genetic interactions and solid lines represent protein-protein interactions. Arrowheads represents the directionality of the interaction with solid squares representing activators, open squares representing enzymatic activity, diamonds representing genetic interactors, solid arrows representing physical interactors, solid circles representing products, the “T” representing repressors, the “C” representing substrates, the dashed solid arrow representing transcribed genes and the dashed open arrow representing transcription factors.

**Fig 6:**
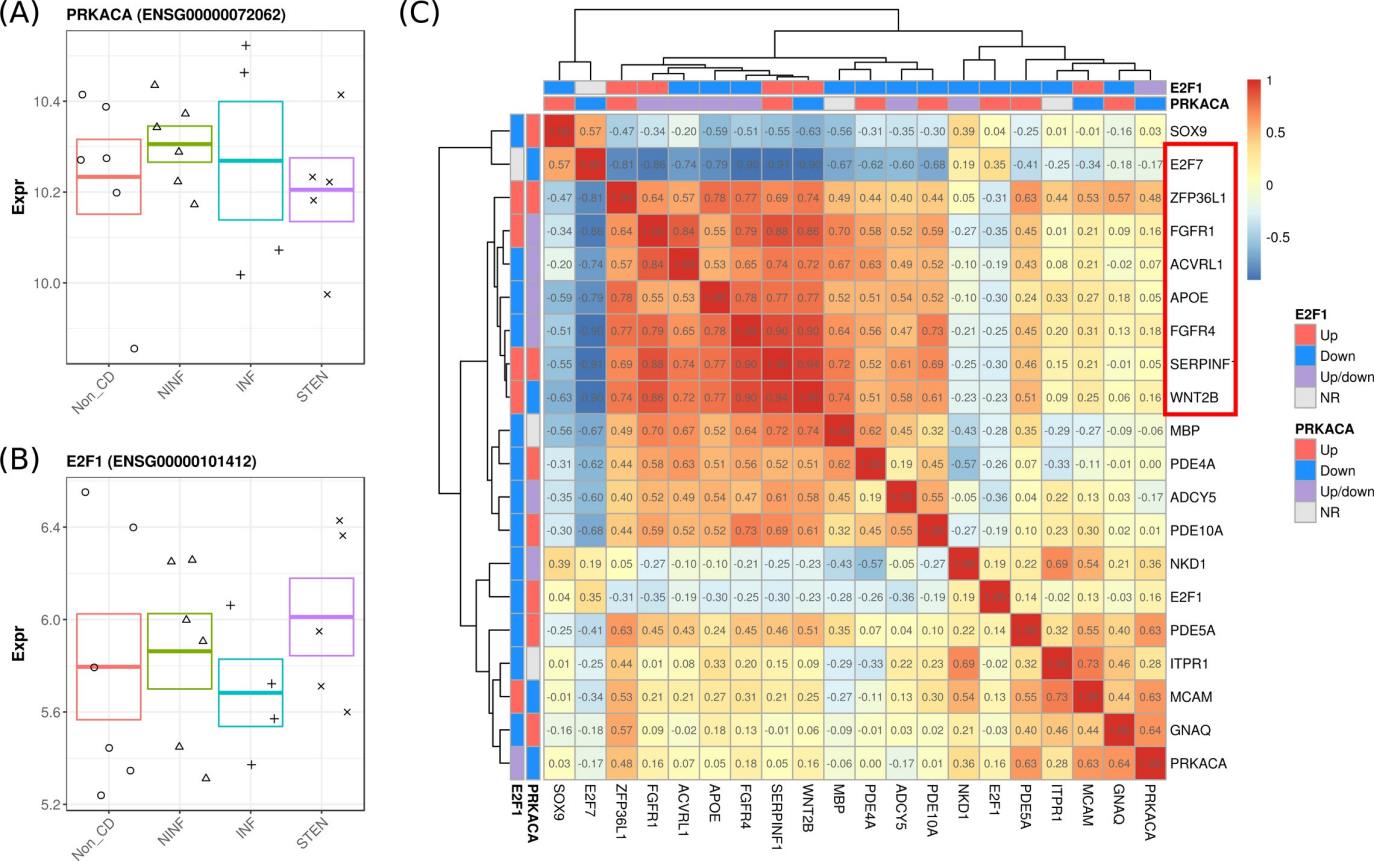
Overview of the *PRKACA/E2F1*-associated genes. Jitter plot representing the gene expression for the highly-interconnected genes (A) *PRKACA* and (B) *E2F1*. Error bars represent the standard error. (C) Heatmap representing the pairwise gene correlation coefficients for the genes linked to *PRKACA* and E2F1. Colored bars above the heatmap represent the relationship of the genes with *PRKACA* and *E2F1*, where upstream genes (‘Up’) are represented in red, downstream genes (‘Down’) in blue, up and downstream genes (‘Up/down’) in purple, and genes with no relationship (‘NR’) in grey. Highlighted in red are the genes whose absolute inter-gene correlations are higher than 0.7.

To understand whether the DMEGs were interconnected, we performed an induced network analysis (Fig 5). Overall, two genes were observed that appeared to interact with most DMEGs, namely protein kinase CAMP-activated catalytic subunit alpha (*PRKACA*) and E2F transcription factor 1 (*E2F1*). Interestingly, *PRKACA* or *E2F1* were neither differentially methylated nor differentially expressed (Fig 6A). We therefore investigated whether the genes associated to *PRKACA* and *E2F1* were co-expressed. Pairwise correlation analyses suggested strong correlations for 9 genes, namely Wnt family member 2B (*WNT2B*), Serpin Family F Member 1 (*SERPINF1*), Myelin Basic Protein (*MBP*), Fibroblast Growth Factor Receptor 4 (*FGFR4*), apolipoprotein E (*APOE*), Activin A Receptor Like Type 1 (*ACVRL1*), Fibroblast Growth Factor Receptor 1 (*FGFR1*), Zinc Ring Finger Protein Like 1 (*ZFP36L1*), E2F transcription factor 7 (*E2F7*) (Figs 6B and S4). The strongest positive gene-gene correlations were observed primarily among *FGFR4*, *SERPINF1*, and *WNT2B*, whereas a strong inverse correlation was observed for *E2F7* (Table 3).

**Table 3:** PRKACA/E2F1-associated DMEGs.

Coordinates	nCpGs	Gene	Ensembl	Entrez	Correlation	p_correlation	p_DE	p_DM
chr1:113054687–113056390	6	WNT2B	ENSG00000134245	7482	-0.88 [-0.97, -0.39]	0.0583	0.000221	0.006753
chr12:52299524–52301970	10	ACVRL1	ENSG00000139567	94	-0.92 [-0.99, -0.63]	0.0377	9.02E-06	2.15E-05
chr12:77771462–77773503	5	E2F7	ENSG00000165891	144455	-0.8 [-0.92, -0.29]	0.0686	8.21E-08	0.018861
chr14:69052728–69053361	3	ZFP36L1	ENSG00000185650	677	-0.18 [-0.91, 0.65]	0.7015	0.000153	0.009438
chr17:1664329–1666607	10	SERPINF1	ENSG00000132386	5176	-0.88 [-0.99, -0.43]	0.0504	0.000236	1.75E-06
chr18:74779597–74781719	4	MBP	ENSG00000197971	4155	-0.86 [-0.95, -0.4]	0.0619	0.00042	0.002975
chr19:45407860–45407945	3	APOE	ENSG00000130203	348	-0.78 [-0.96, -0.1]	0.0473	0.00024	0.028636
chr5:176515533–176516968	7	FGFR4	ENSG00000160867	2264	-0.61 [-0.95, 0.35]	0.2519	0.000268	0.00579
chr8:38286241–38288404	6	FGFR1	ENSG00000077782	2260	-0.85 [-0.96, -0.37]	0.058	0.000102	0.024728

Columns represent the coordinates of the DMR, the associated gene name, the number of CpGs encompassed by the DMR, the Ensembl gene ID, the Entrez ID, the methylation-expression Pearson correlation coefficient with the 95% confidence intervals, the *p*-value of the correlation coefficient, the p-value of the DE analysis, and the p-value of the DMR analysis.

## Discussion

In this study, we performed an integrative analysis of DNA methylation and gene expression to understand the intrinsic changes associated to CD-induced fibrosis. To this end, fibroblasts were obtained from ileal tissue at macroscopically-defined distinct stages of CD as well as from non-CD patients. As resection material represents a mixed cell population, we cultured the sample such that only fibroblasts would remain. We acknowledge that growing the samples in culture affects DNA methylation [52] and gene expression [53,54]. However, as all samples were passaged, we corrected for passage by including it as a covariate in the DNA methylation and gene expression analyses.

We observed most differences when comparing STEN with NINF fibroblasts. Conversely, few changes were observed when comparing CD with non-CD, or INF with NINF. We hypothesize however that the limited sample size of the INF cohort precluded us from finding any meaningful results. By performing pathway analyses on the DMRs and DEGs we observed concordant dysregulation for pathways associated to cellular differentiation, morphogenesis, and ECM remodeling. In particular, we observed consistent downregulation and promoter hypermethylation of genes associated with ECM processing and elastic fiber formation, implying systematic downregulation. Surprisingly, ECM-associated genes were found to be upregulated for idiopathic pulmonary- (IPF) [55,56], lung- [57], cardiac- [58], and CD-associated ileal fibrosis [59]. Despite observing similarly affected pathways, we are currently unsure why our results contrast previous findings.

As the methylation and gene expression analyses implicated similar pathways, we integrated the DMRs and the DEGs by overlapping the datasets and performing an eQTM analysis. Through induced network analysis, we identified two genes that interacted with most DMEGs, namely *PRKACA* and *E2F1*. *PRKACA* encodes one of the catalytic subunits of protein kinase A (PKA) and plays an important role in meiosis [60,61] and glucose metabolism [62]. Given its role in several pathways, dysregulation of PKA has been associated with multiple disorders including cardiovascular diseases [63], tumor formation [64], and fibrolamellar hepatocellular carcinoma [65]. Interestingly, no difference in *PRKACA* gene expression was observed across the different phenotypes. E2F1 is a transcription factor belonging to the family of E2F transcription factors. Despite the lack of statistical significance, our data suggested an increase in the level of *E2F1* gene expression when comparing STEN with non-CD and NINF fibroblasts. Recent studies on cholestatic liver fibrosis observed an increase in the gene expression of *E2F1*, where it was found to act as a transcription factor alongside nuclear receptor small heterodimer partner (NR0B2; SHP) and co-repressor EP300 Interacting Inhibitor Of Differentiation 1 (EID1) in controlling the gene expression of Early Growth Response 1 (*EGR1*) [66], the latter of which has been implicated in the development of fibrosis [67–71].

Investigation of the DMEGs associated to *PRKACA/E2F1* revealed strong co-expression. Moreover, the methylation and the expression of the DMEGs were strongly correlated suggesting concordant dysregulation. Many of the *PRKACA/E2F1*-associated genes have been implicated in fibrotic processes previously. *SERPINF1* (S4B Fig), also known as pigment epithelium-derived factor (*PEDF*), was found to exhibit anti-angiogenic properties [72] by cleaving the vascular endothelial growth factor (VEGF) receptor 1 [73]. Our data indicated that *SERPINF1* expression was downregulated in stenotic fibroblasts. Conversely, E2F7 (S4I Fig) was found to promote angiogenesis through transcriptional activation of *VEGFA* [74] and was upregulated in stenotic fibroblasts, which corroborates previous findings in mice where *E2F7* was found to be upregulated in renal fibrotic tissue [75]. Taken together, the observed downregulation of *SERPINF1* and upregulation of *E2F7* could promote angiogenesis, which is a characteristic of IBD [76,77] and fibrosis [78].

Just like *E2F1*, *E2F7* belongs to the family of E2F transcription factors, which play a central role in a wide range of biological processes such as differentiation, cell division [79], and DNA repair [80]. E2F transcription factors interact with several fibroblast growth factor receptors by regulating *FGFR1* (S4G Fig) [81] and *FGFR2* [82]. FGFR1 suppresses TGFβ and mitogen-activated protein kinase kinase kinase kinase 4 (MAP4K4) [83], which is involved in endothelial-mesenchymal transition (EndoMT). Excessive EndoMT was found to contribute to cardiac [84–86] and idiopathic lung fibrosis [87]. Downregulation of *FGFR1* could therefore induce EndoMT, which could contribute towards the observed fibrotic phenotype [86]. Interestingly, the opposite effect was observed for *FGFR4 (*S4D Fig), whose gene expression was downregulated in stenotic fibroblasts. In hepatocellular carcinoma samples, *FGFR4* expression was found to be increased alongside the expression of *TGFβ* [88], suggesting that an increase in expression contributes towards fibrosis.

Besides the regulation of FGF pathways, E2F transcription factors also affect WNT pathways [89] primarily through E2F1, which was found to suppress Wnt/β-catenin signaling pathway by activating inhibitor of β-catenin and TCF4 (ICAT) [90]. Moreover, Wnt signaling was found to induce epithelial to mesenchymal transition, which, just like EndoMT, is a contributing factor of fibrosis [86]. Among the *PRKACA/E2F1* genes, we observed downregulation of Wnt signaling pathway member *WNT2B*, whose expression was correlated with an increase in methylation (S4A Fig). As IBD-associated mutations of constituent genes within the Wnt signaling pathway are rare, it was speculated that the epigenetic landscape surrounding Wnt-related genes were likely affected in CD [91]. Our data alongside others’ [24,92] show that the expression of *WNT2B* is downregulated in STEN relative to NINF fibroblasts, which correlates with an increased methylation signal. STAT6-deficient mice, which display a delayed wound-healing phenotype akin to fibrosis, displayed diminished gene expression of *Wnt2b*, *Wnt7b*, and *Wnt10a* after 2,4,6-Trinitrobenzenesulfonic acid (TNBS) treatment [92]. Moreover, STAT6-deficient mice treated with TNBS displayed diminished accumulation of the classical WNT signaling protein β-catenin in the nucleus of mucosal cells relative to TNBS-treated control mice corroborating the impaired gene expression of *Wnt2b* [92].

Altogether, we observe that multiple components of the interconnected E2F, WNT, and FGF pathways [93] are dysregulated at the level of DNA methylation and gene expression. As the E2F, WNT and FGF pathways are important for cellular differentiation, proliferation, and migration, aberrant behavior thereof could underlie tissue remodeling processes, resulting in the observed fibro-stenotic phenotype [94–96].

Similar methylomic and transcriptomic changes were observed for genes encoding proteins that are involved in lipid metabolism. *APOE* was found to be downregulated, which was correlated with an increase in methylation (S4E Fig). *APOE*-deficient mice were found to develop metabolic syndromes, non-alcoholic steatohepatitis and liver fibrosis after being fed a cholesterol and fat rich diet or a methionine-choline-deficient-diet (MCD) [97]. Similarly, we observed *ZFP36L1* to be downregulated (S4H Fig), which regulates Cytochrome P450 Family 7 Subfamily A Member 1 (*CYP7A1*) mRNA [98]. CYP7A1 is a rate limiting enzyme involved in cholesterol metabolism into bile acid [99]. Downregulation of *ZFP36L1* can therefore increase bile production leading to bile acid malabsorption, which is a common sign of IBD [100].

By performing integrated analyses of the methylome and the transcriptome we have observed changes in pathways associated to ECM processes and lipid metabolism. By narrowing our search to genes that were affected at the level of methylation and expression, we extracted novel targets for future studies. Nonetheless, further research is needed to validate the aforementioned results, where it would be important to investigate whether protein expression of the DMEGs corroborates the observed transcriptional and methylomic changes. Furthermore, additional research is necessary to understand the directionality of the methylation-expression relationship to ascertain causality. In particular, it would be worthwhile to investigate the effect and possibility of rectifying the aberrant methylation pattern, which would provide us with more insight into the intrinsic mechanisms underlying CD-associated fibrosis.

## Supporting information

S1 Fig Principal component analysis methylation and expression. Correlation of the degree and passage with each principal component for the (A) methylation data and (B) expression data.
(PDF)

S2 Fig Volcano plot of the CpG loci on the Illumina EPIC BeadChip array. The y-axis depicts the statistical significance (-log_10_(*p*-value)) and x-axis depicts the percentage difference in methylation. (A) CD versus non-CD, (B) INF versus NINF, and (C) STEN vs NINF. Colors indicate whether a CpG is differentially methylated with a large effect size (Beta > 0.2; ‘sig. interesting’), differentially methylated with a small effect size (‘significant’), or not differentially methylated (‘non-significant’). The top 10 DMPs are labelled with their Illumina probe ID.
(PDF)

S3 Fig Volcano plot of the genes measured through RNAseq experiment. The y-axis depicts the statistical significance (-log_10_(p-value)) and the x-axis the log_2_ fold change. (A) CD versus non-CD, (B) INF versus NINF, and (C) STEN vs NINF. Colors indicate whether a gene is differentially expressed with a large effect size (logFC > 1; ‘sig. interesting’), differentially expressed with a small effect size (‘significant’), or not differentially expressed (‘non-significant’). The top 10 DMPs are labelled with their Ensembl gene ID.
(PDF)

S4 Fig Summary plots of the DMEGs. (A) WNT2B, (B) SERPINF1, (C) MBP, (D) FGFR4, (E) APOE, (F) ACVRL1, (G) FGFR1, (H) ZFP36L1, and (I) E2F7. At the top: Genomic coordinates at the top represent the chromosome and the genomic positions of the DMRs with the gene of interest highlighted in red. Bottom left: Detailed genome plot of the methylation values of the CpGs that comprise the DMR of interest. Bottom middle: Plot of the expression representing the mean log-transformed expression and the standard error. Bottom right: Correlation plot of the log transformed expression on the x-axis against the mean methylation (Beta) of the DMR on the y-axis for the 9 samples present in both the methylation and expression experiment.
(PDF)

S1 Table Overlapping samples. An overview of all the samples included in the methylation and expression studies. Details include the sample, patient, and passage number. Samples that were included in both the methylation and expression study are shown in bold text.
(XLSX)

S2 Table Differentially methylated regions. Differentially methylated regions as found for CD vs non-CD (Tab 1), CD-inflamed vs CD-non-inflamed (Tab 2), and CD-stenotic vs CD-non-inflamed (Tab 3). Columns represent the coordinates of the DMR, the number of CpGs encompassed, the Stouffer statistic that represents a summary significance statistic of the DMR, the mean methylation signal across the DMR, the Ensembl gene ID, the gene symbol, and a gene name.
      Tab 1: CD vs non-CD.
      Tab 2: INF vs NINF.
      Tab 3: STEN vs NINF.
(XLSX)

S3 Table DMR pathway enrichment. Columns represent the database, the gene set ID, the description of the gene set, the enrichment *p*-value, the BH-adjusted *p*-value, the number of genes within the set, the number of observed genes from the set, the average gene length of the genes within the set, the Entrez IDs for the genes located in the gene set.
      Tab 1: CD vs non-CD hypermethylated DMRs.
      Tab 2: CD vs non-CD hypomethylated DMRs.
      Tab 3: STEN vs NINF hypermethylated DMRs.
      Tab 4: STEN vs NINF hypomethylated DMRs.
(XLSX)

S4 Table Differentially expressed genes. Differentially expressed genes as found for the CD vs non-CD (Tab 1), CD-inflamed vs CD-non-inflamed (Tab 2), and CD-stenotic vs CD-non-inflamed (Tab 3). Columns represent the log_*2*_ fold change, the standard error of the log_*2*_ fold change, the Wald statistic, the *p-*value, the BH-adjusted *p-*value, the gene symbol, the Entrez ID, and the Ensembl gene ID.
      Tab 1: CD vs non-CD.
      Tab 2: INF vs NINF.
      Tab 3: STEN vs NINF.
(XLSX)

S5 Table DEG gene set enrichment. Columns represent the database, the gene set ID, the description of the gene set, the enrichment *p*-value, the BH-adjusted *p*-value, the number of genes within the set, the number of observed genes from the set, the average gene length of the genes within the set, the Entrez IDs for the genes located in the gene set.
      Tab 1: CD vs non-CD upregulated genes.
      Tab 2: CD vs non-CD downregulated genes.
      Tab 3: STEN vs NINF upregulated genes.
      Tab 4: STEN vs NINF downregulated genes.
(XLSX)

S6 Table DMEGs: Differentially methylated and expressed genes as found for STEN vs NINF. Columns represent the coordinates of the DMR, the associated gene name, the number of CpGs encompassed by the DMR, the Ensembl gene ID, the Entrez ID, the methylation-expression Pearson correlation coefficient alongside the 95% confidence intervals, the *p*-value of the correlation coefficient, the *p*-value of the DE analysis, and the *p*-value of the DMR analysis.
(XLSX)

S7 Table DMEGs gene set overrepresentation analyses against all the genes measured as measured by CPDB.       Tab 1: Network neighborhood-based entity sets (NESTs). Columns represent the *p*-value, the BH-adjusted *p-*value, the name of the center, the Entrez ID of the NEST, the size of the NEST, the effective size of the NEST, the Ensembl gene IDs of the DMEGs within the NEST, and the sources of the NEST.
      Tab 2: Pathway-based sets. Columns represent the *p*-value, the BH-adjusted *p-*value, the source database, the ID, the size of the gene set, the effective size of the gene set, and the Ensembl gene IDs of the DMEGs within the gene set.
(XLSX)

## Abbreviations

CD: Crohn’s disease
IBD: Inflammatory bowel disease
GWAS: Genome-wide association study
EWAS: Epigenome-wide association study
ECM: Extracellular matrix
NINF: CD Non-inflamed tissue
INF: CD Inflamed tissue
STEN: CD Stenotic tissue
CpG: Cytosine followed by a guanine
DMP: Differentially methylated position
DMR: Differentially methylated region
DEG: Differentially expressed gene
DMEG: Differentially methylated and expressed gene
eQTM: Expression quantitative trait methylation
TSS: Transcription start site
