# Supplementary figures and images for: A distinct epigenetic profile distinguishes stenotic from non-inflamed fibroblasts in the ileal mucosa of Crohn’s disease patients
Source: PLoS One. 2018 Dec 27;13(12):e0209656. doi: 10.1371/journal.pone.0209656 (PMC6307755; doi:10.1371/journal.pone.0209656)

(A)

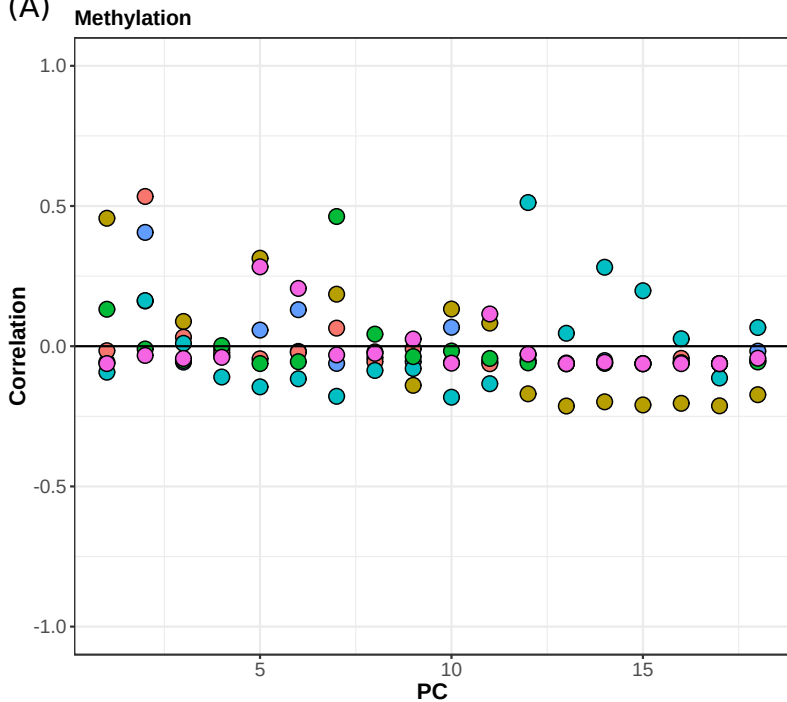

(B)

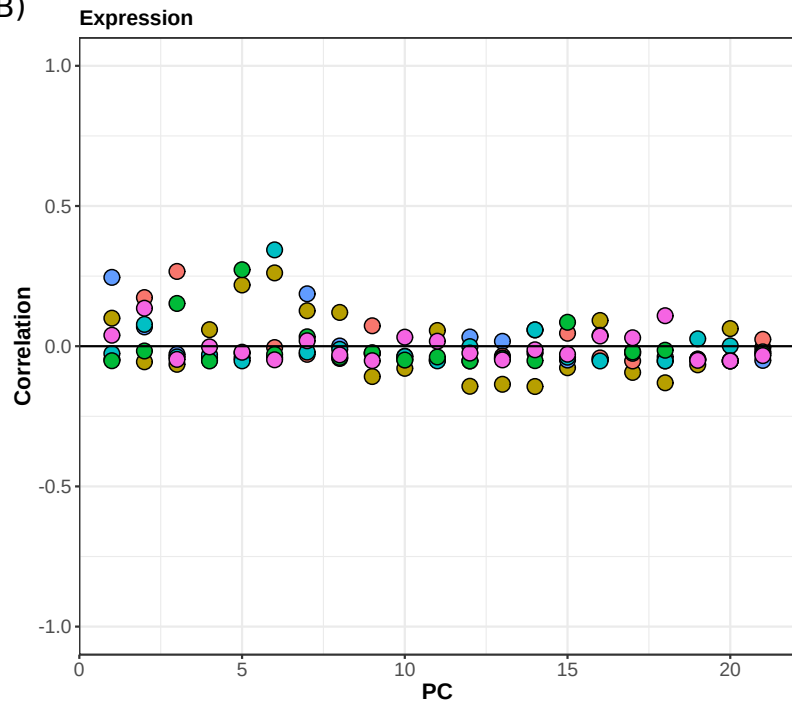

Supplement: S1 Fig — Correlation of the degree and passage with each principal component for the (A) methylation data and (B) expression data. (PDF) [file pone.0209656.s001.pdf]

Figure S2

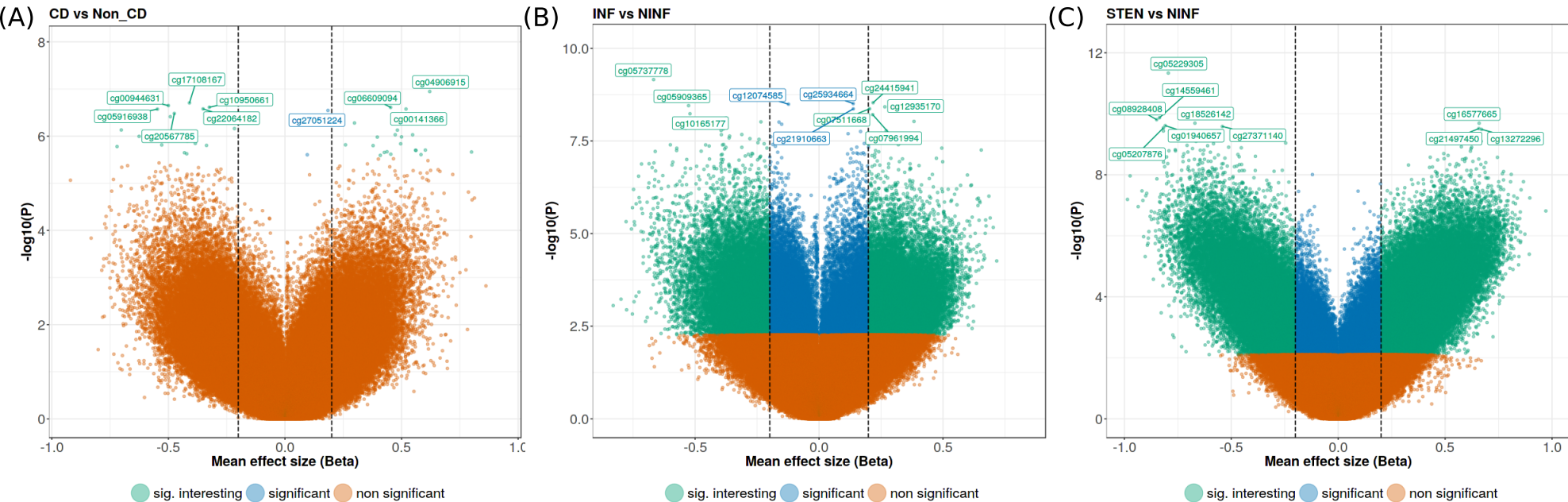

Supplement: S2 Fig — The y-axis depicts the statistical significance (-log10(p-value)) and x-axis depicts the percentage difference in methylation. (A) CD versus non-CD, (B) INF versus NINF, and (C) STEN vs NINF. Colors indicate whether a CpG is differentially methylated with a large effect size (Beta > 0.2; ‘sig. interesting’), differentially methylated with a small effect size (‘significant’), or not differentially methylated (‘non-significant’). The top 10 DMPs are labelled with their Illumina probe ID. (PDF) [file pone.0209656.s002.pdf]

Figure S3

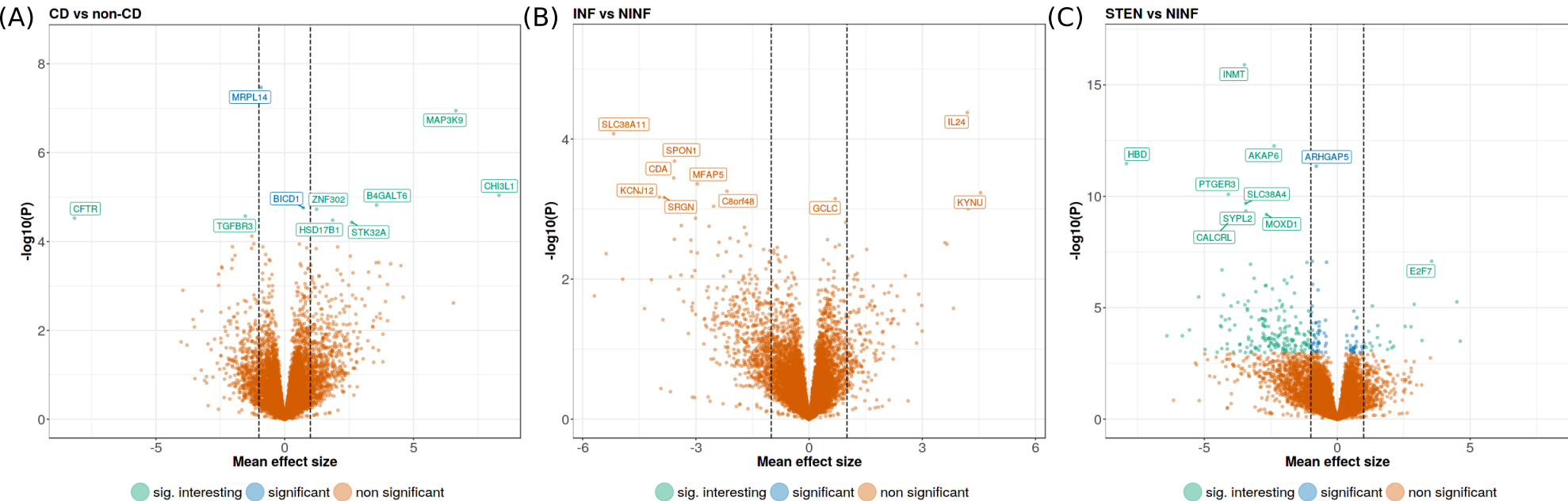

Supplement: S3 Fig — The y-axis depicts the statistical significance (-log10(p-value)) and the x-axis the log2 fold change. (A) CD versus non-CD, (B) INF versus NINF, and (C) STEN vs NINF. Colors indicate whether a gene is differentially expressed with a large effect size (logFC > 1; ‘sig. interesting’), differentially expressed with a small effect size (‘significant’), or not differentially expressed (‘non-significant’). The top 10 DMPs are labelled with their Ensembl gene ID. (PDF) [file pone.0209656.s003.pdf]
