# Supplementary material for: A distinct epigenetic profile distinguishes stenotic from non-inflamed fibroblasts in the ileal mucosa of Crohn’s disease patients
Source: PLoS One. 2018 Dec 27;13(12):e0209656. doi: 10.1371/journal.pone.0209656 (PMC6307755; doi:10.1371/journal.pone.0209656)
Supplement: S4 Fig — (A) WNT2B, (B) SERPINF1, (C) MBP, (D) FGFR4, (E) APOE, (F) ACVRL1, (G) FGFR1, (H) ZFP36L1, and (I) E2F7. At the top: Genomic coordinates at the top represent the chromosome and the genomic positions of the DMRs with the gene of interest highlighted in red. Bottom left: Detailed genome plot of the methylation values of the CpGs that comprise the DMR of interest. Bottom middle: Plot of the expression representing the mean log-transformed expression and the standard error. Bottom right: Correlation plot of the log transformed expression on the x-axis against the mean methylation (Beta) of the DMR on the y-axis for the 9 samples present in both the methylation and expression experiment. (PDF) [file pone.0209656.s004.pdf]

(A) WNT2B

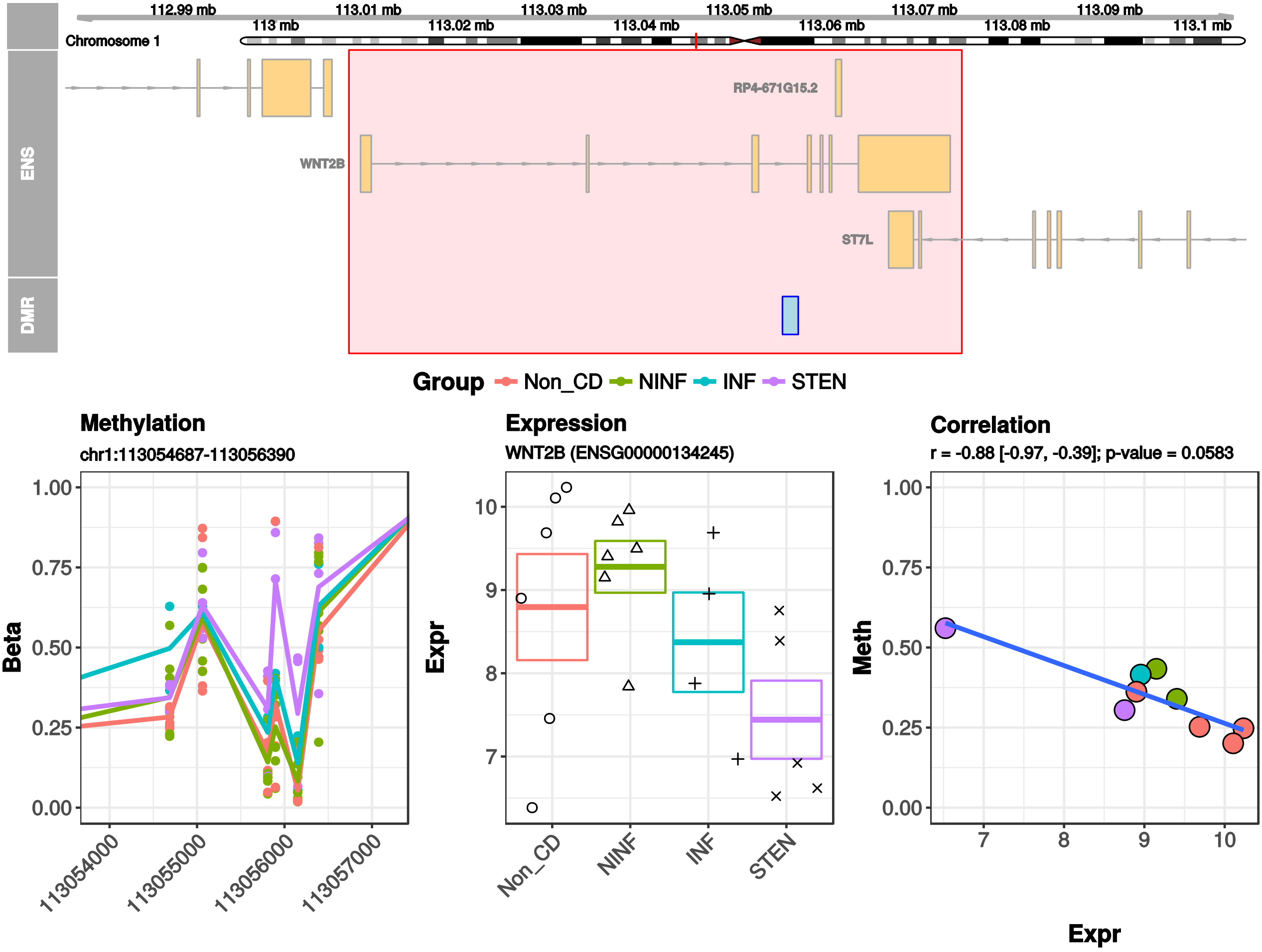

112.99 mb
113 mb
113.01 mb
113.02 mb
113.03 mb
113.04 mb
113.05 mb
113.06 mb
113.07 mb
113.08 mb
113.09 mb
113.1 mb
Chromosome 1
ENS
DMR
RP4-671G15.2
WNT2B
ST7L
Group
Non_CD
NINF
INF
STEN
Methylation
chr1:113054687-113056390
Beta
1.00
0.75
0.50
0.25
0.00
113054000
113055000
113056000
113057000
Expression
WNT2B (ENSG00000134245)
Expr
10
9
8
7
Non_CD
NINF
INF
STEN
Correlation
r = -0.88 [-0.97, -0.39]; p-value = 0.0583
Meth
1.00
0.75
0.50
0.25
0.00
7
8
9
10
Expr

(B) SERPINF1

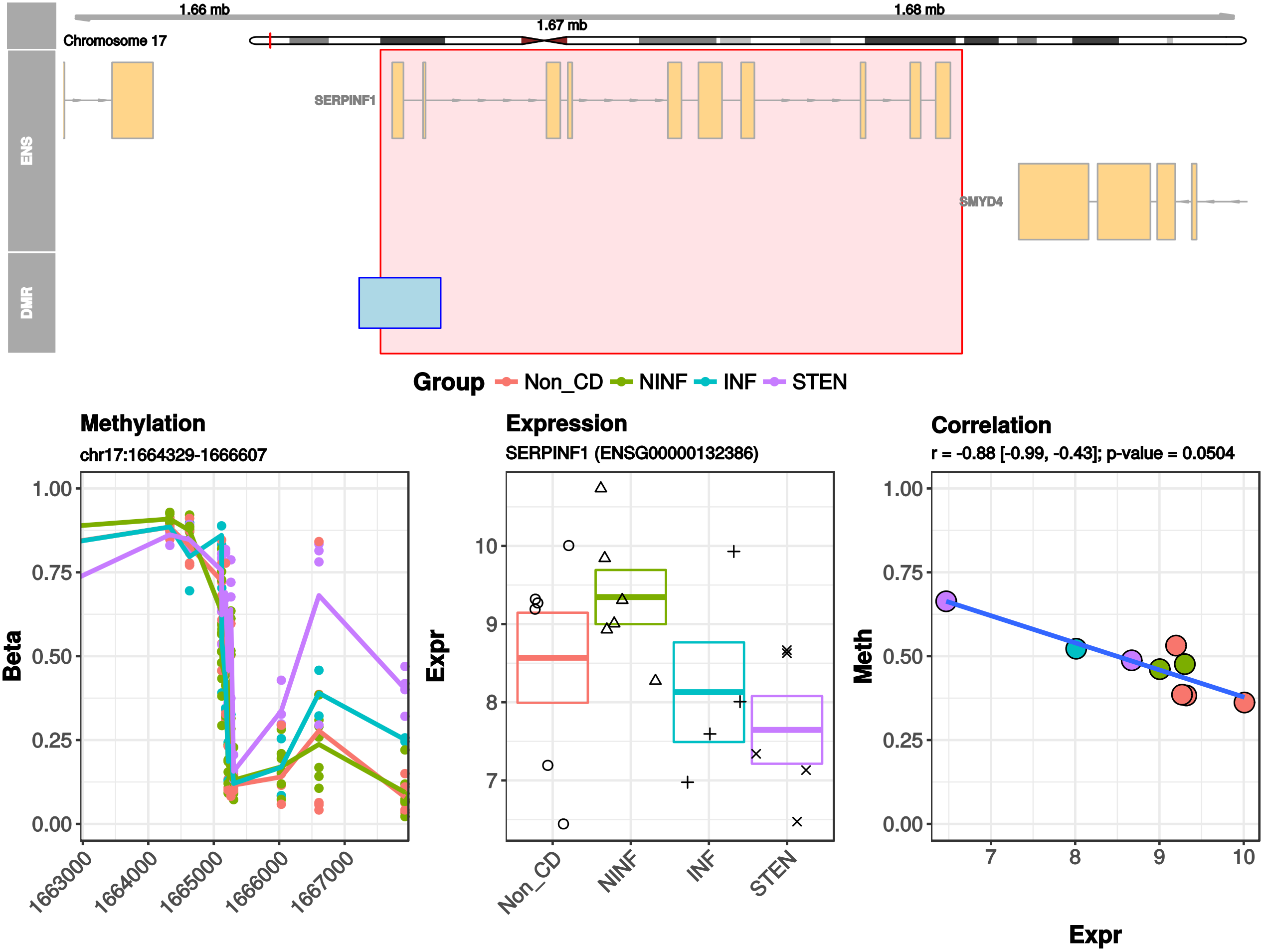

1.66 mb
1.67 mb
1.68 mb
Chromosome 17
ENS
DMR
SERPINF1
SMYD4
Group
Non_CD
NINF
INF
STEN
Methylation
chr17:1664329-1666607
Beta
Expression
SERPINF1 (ENSG00000132386)
Expr
Correlation
r = -0.88 [-0.99, -0.43]; p-value = 0.0504
Meth
Expr

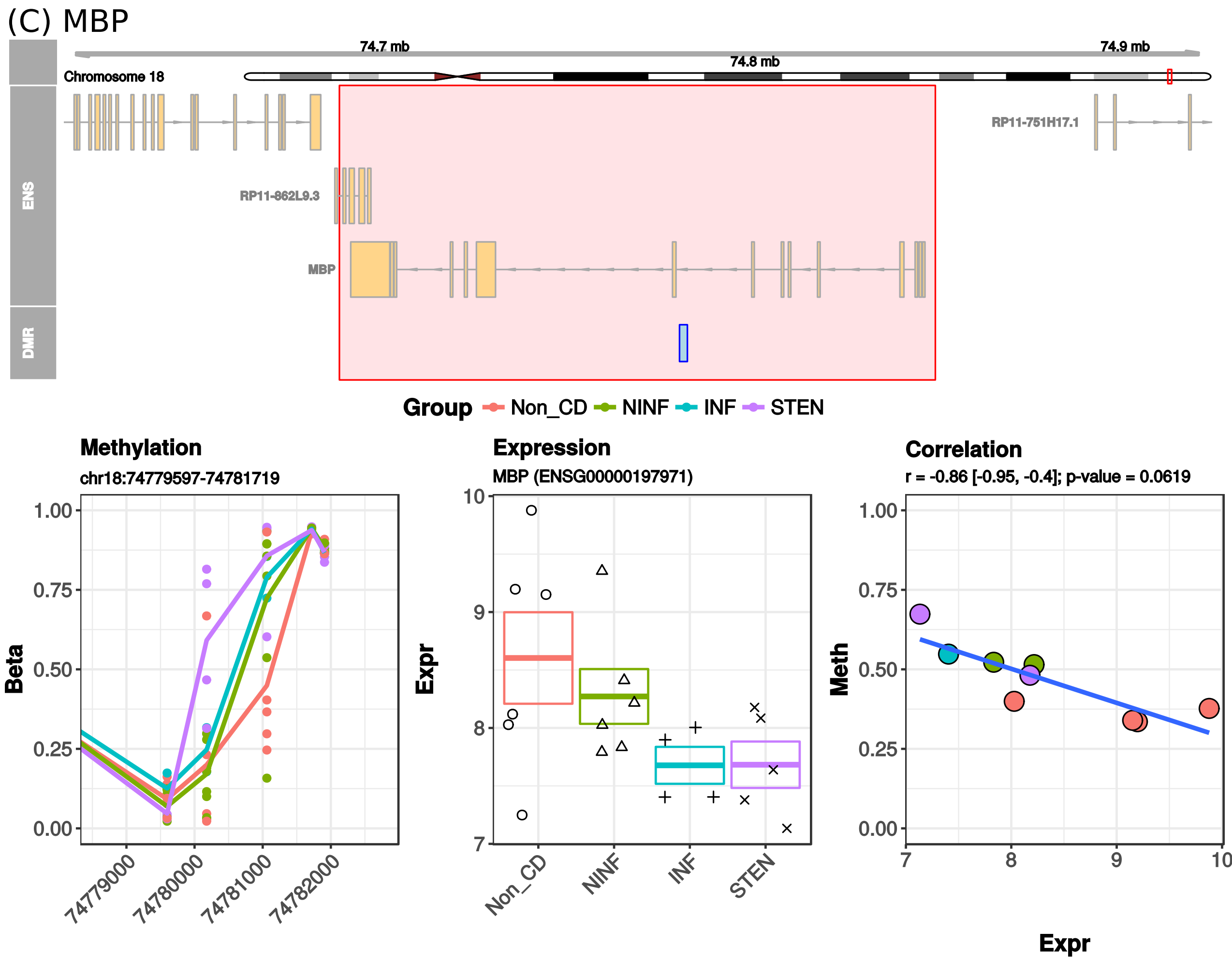
(C) MBP
74.7 mb
74.8 mb
74.9 mb
Chromosome 18
ENS
DMR
RP11-862L9.3
MBP
RP11-751H17.1
Group Non_CD NINF INF STEN
Methylation
chr18:74779597-74781719
Beta
1.00
0.75
0.50
0.25
0.00
74779000
74780000
74781000
74782000
Expression
MBP (ENSG00000197971)
Expr
10
9
8
7
Non_CD
NINF
INF
STEN
Correlation
r = -0.86 [-0.95, -0.4]; p-value = 0.0619
Meth
Expr
7
8
9
10

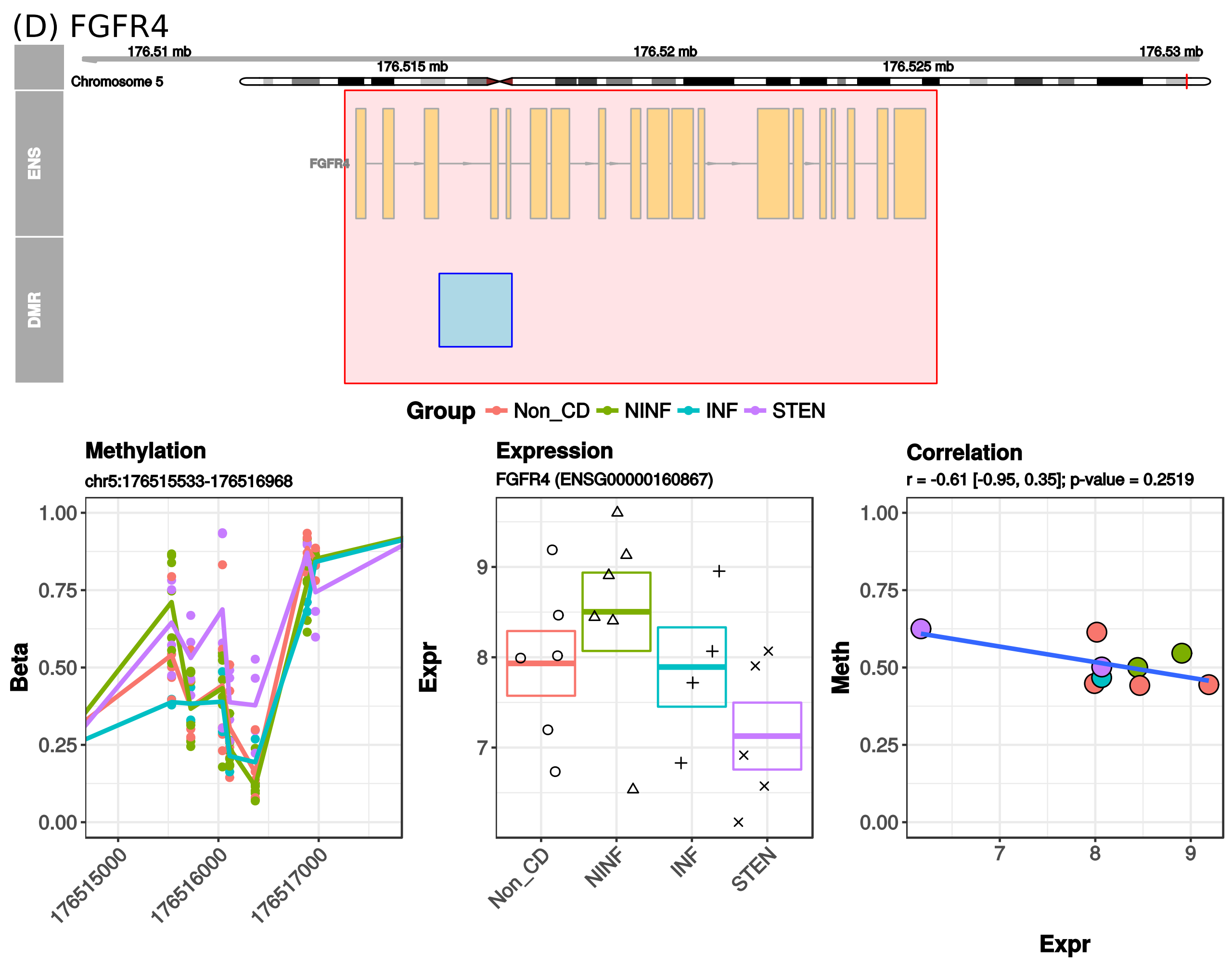

(D) FGFR4
176.51 mb
176.515 mb
176.52 mb
176.525 mb
176.53 mb
Chromosome 5
ENS
FGFR4
DMR
Group
Non_CD
NINF
INF
STEN
Methylation
chr5:176515533-176516968
Beta
1.00
0.75
0.50
0.25
0.00
176515000
176516000
176517000
Expression
FGFR4 (ENSG00000160867)
Expr
9
8
7
Non_CD
NINF
INF
STEN
Correlation
r = -0.61 [-0.95, 0.35]; p-value = 0.2519
Meth
1.00
0.75
0.50
0.25
0.00
7
8
9
Expr

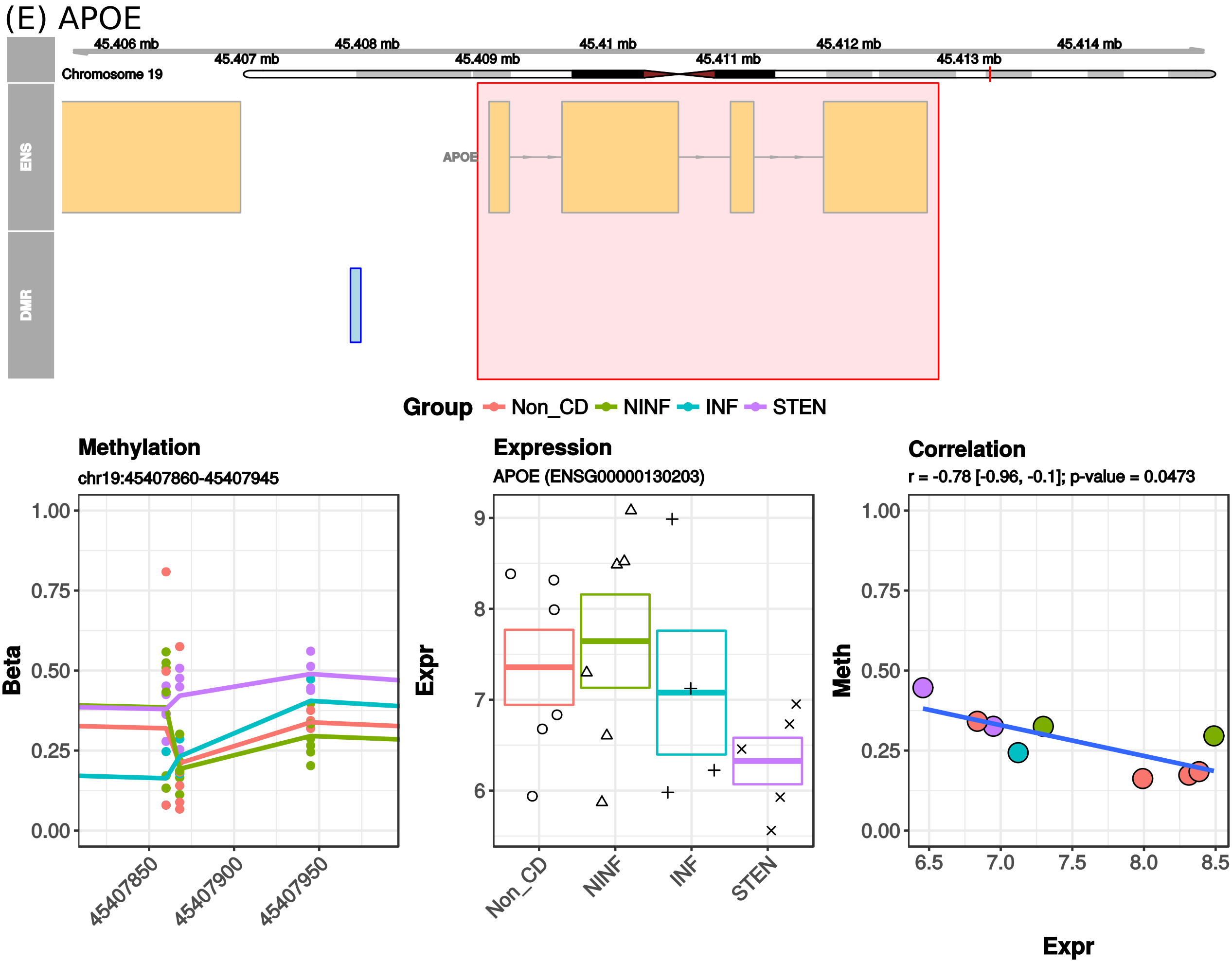

(E) APOE
45.406 mb
45.407 mb
45.408 mb
45.409 mb
45.41 mb
45.411 mb
45.412 mb
45.413 mb
45.414 mb
Chromosome 19
ENS
APOE
DMR
Group Non_CD NINF INF STEN
Methylation
chr19:45407860-45407945
Beta
1.00
0.75
0.50
0.25
0.00
45407850
45407900
45407950
Expression
APOE (ENSG00000130203)
Expr
9
8
7
6
Non_CD
NINF
INF
STEN
Correlation
r = -0.78 [-0.96, -0.1]; p-value = 0.0473
Meth
1.00
0.75
0.50
0.25
0.00
6.5
7.0
7.5
8.0
8.5
Expr

(F) ACVRL1

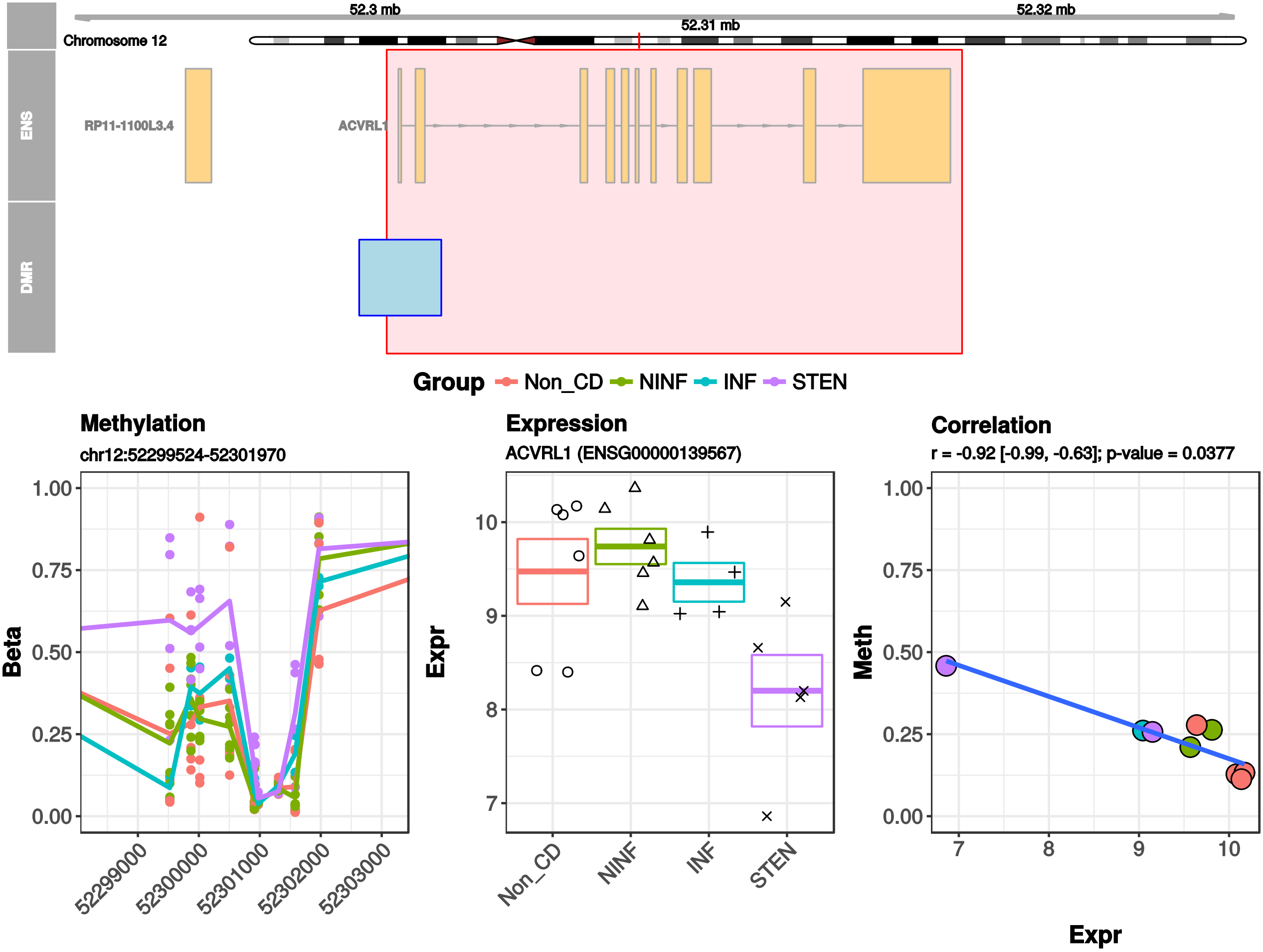
52.3 mb
52.31 mb
52.32 mb
Chromosome 12
ENS
RP11-1100L3.4
ACVRL1
DMR
Group
Non_CD
NINF
INF
STEN
Methylation
chr12:52299524-52301970
Beta
1.00
0.75
0.50
0.25
0.00
52299000
52300000
52301000
52302000
52303000
Expression
ACVRL1 (ENSG00000139567)
Expr
10
9
8
7
Non_CD
NINF
INF
STEN
Correlation
r = -0.92 [-0.99, -0.63]; p-value = 0.0377
Meth
1.00
0.75
0.50
0.25
0.00
7
8
9
10
Expr

(G) FGFR1

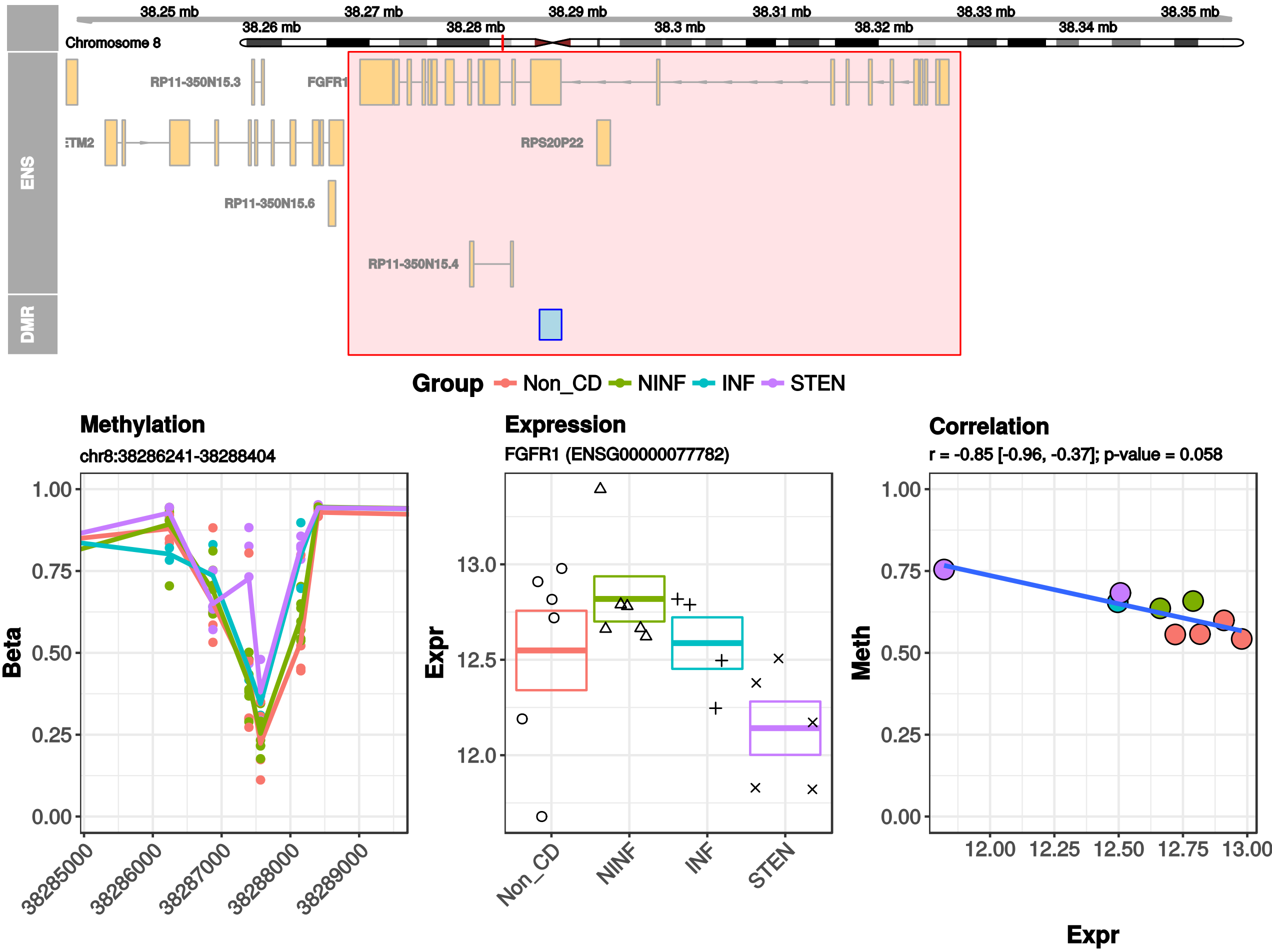

ENS
DMR
Chromosome 8
38.25 mb
38.26 mb
38.27 mb
38.28 mb
38.29 mb
38.3 mb
38.31 mb
38.32 mb
38.33 mb
38.34 mb
38.35 mb
RP11-350N15.3
FGFR1
TM2
RPS20P22
RP11-350N15.6
RP11-350N15.4
Group
Non_CD
NINF
INF
STEN
Methylation
chr8:38286241-38288404
Beta
1.00
0.75
0.50
0.25
0.00
38285000
38286000
38287000
38288000
38289000
Expression
FGFR1 (ENSG00000077782)
Expr
13.0
12.5
12.0
Non_CD
NINF
INF
STEN
Correlation
r = -0.85 [-0.96, -0.37]; p-value = 0.058
Meth
1.00
0.75
0.50
0.25
0.00
12.00
12.25
12.50
12.75
13.00
Expr

(H) ZFP36L1

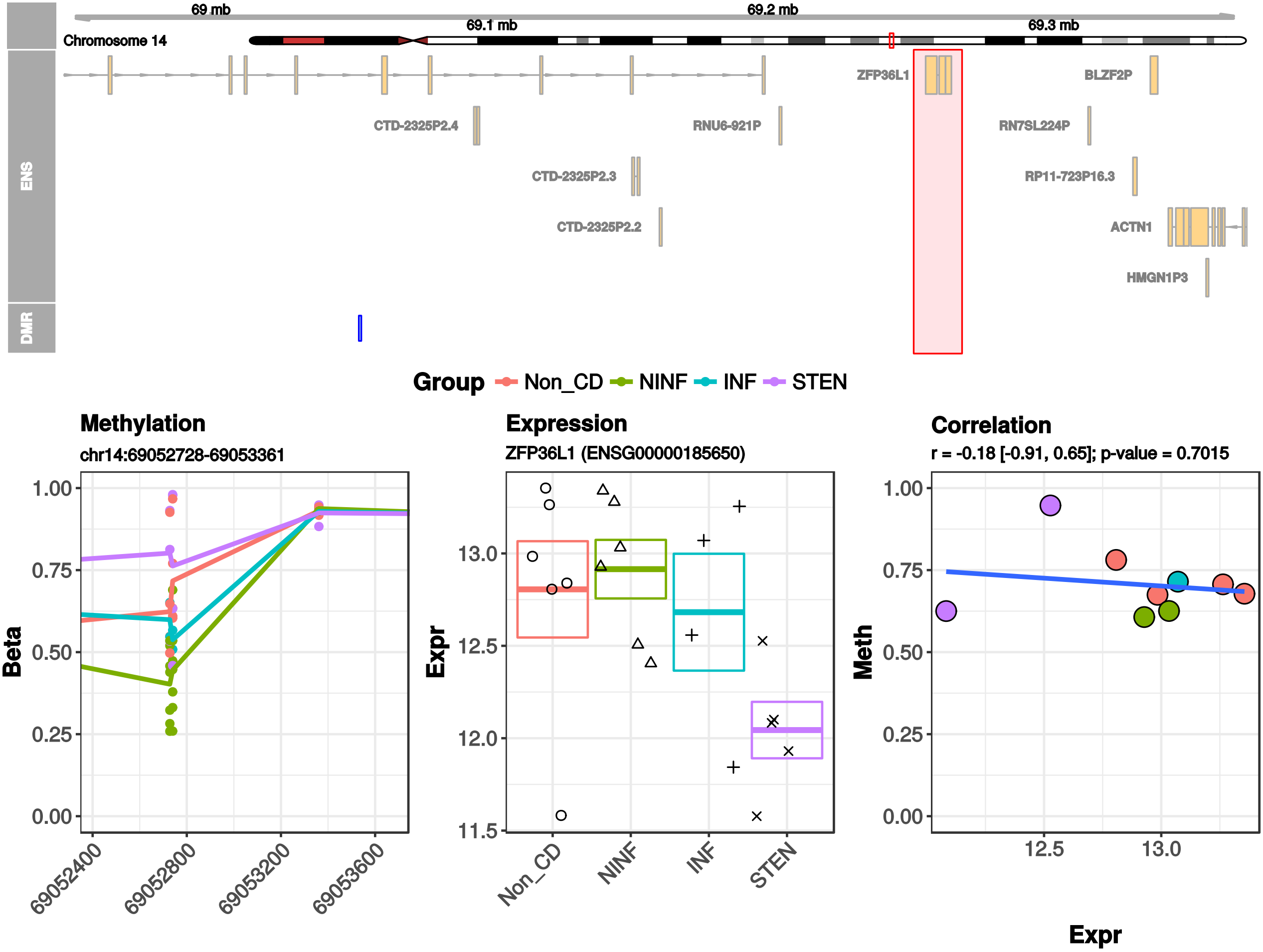

69 mb
69.1 mb
69.2 mb
69.3 mb
Chromosome 14
ENS
DMR
ZFP36L1
BLZF2P
CTD-2325P2.4
RNU6-921P
RN7SL224P
CTD-2325P2.3
RP11-723P16.3
CTD-2325P2.2
ACTN1
HMGN1P3
Group Non_CD NINF INF STEN
Methylation
chr14:69052728-69053361
Beta
Expression
ZFP36L1 (ENSG00000185650)
Expr
Non_CD
NINF
INF
STEN
Correlation
r = -0.18 [-0.91, 0.65]; p-value = 0.7015
Meth
Expr

(I) E2F7

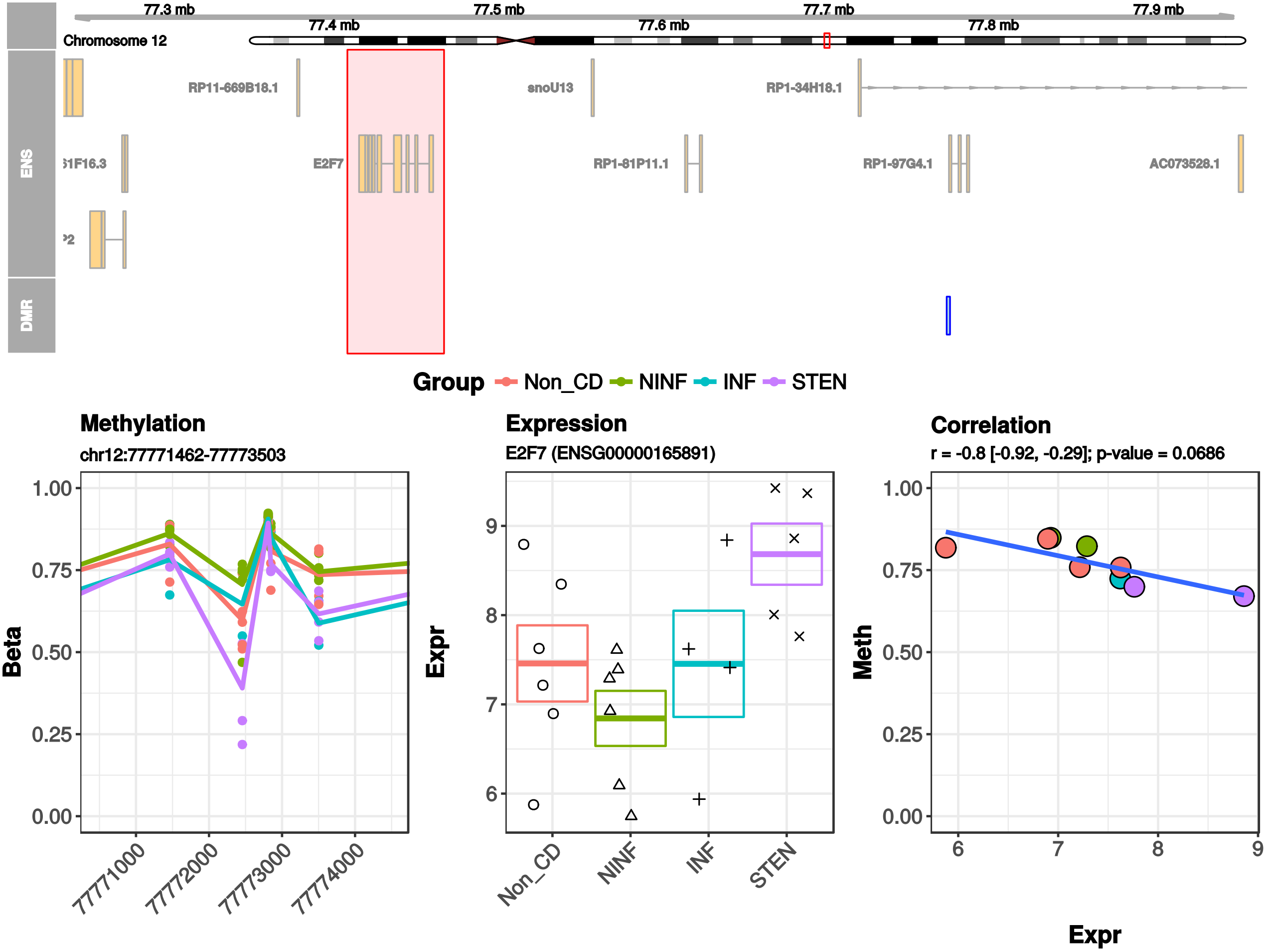

77.3 mb
77.4 mb
77.5 mb
77.6 mb
77.7 mb
77.8 mb
77.9 mb
Chromosome 12
ENS
DMR
RP11-669B18.1
snoU13
RP1-34H18.1
E2F7
RP1-81P11.1
RP1-97G4.1
AC073528.1
Group
Non_CD
NINF
INF
STEN
Methylation
chr12:77771462-77773503
Beta
Expression
E2F7 (ENSG00000165891)
Expr
Correlation
r = -0.8 [-0.92, -0.29]; p-value = 0.0686
Meth
Expr
